# A farnesyl-dependent structural role for CENP-E in expansion of the fibrous corona

**DOI:** 10.1083/jcb.202303007

**Published:** 2023-11-07

**Authors:** Jingchao Wu, Maximilian W.D. Raas, Paula Sobrevals Alcaraz, Harmjan R. Vos, Eelco C. Tromer, Berend Snel, Geert J.P.L. Kops

**Affiliations:** 1 Hubrecht Institute, Royal Netherlands Academy of Arts and Sciences, Utrecht, Netherlands; 2 University Medical Center Utrecht, Utrecht, Netherlands; 3 Oncode Institute, Utrecht, Netherlands; 4 Theoretical Biology and Bioinformatics, Department of Biology, Faculty of Science, Utrecht University, Utrecht, Netherlands; 5 Center for Molecular Medicine, Molecular Cancer Research, University Medical Center Utrecht, Utrecht, Netherlands; 6 Faculty of Science and Engineering, Cell Biochemistry, Groningen Biomolecular Sciences and Biotechnology Institute, University of Groningen, Groningen, Netherlands

## Abstract

Correct chromosome segregation during cell division depends on proper connections between spindle microtubules and kinetochores. During prometaphase, kinetochores are temporarily covered with a dense protein meshwork known as the fibrous corona. Formed by oligomerization of ROD/ZW10/ZWILCH-SPINDLY (RZZ-S) complexes, the fibrous corona promotes spindle assembly, chromosome orientation, and spindle checkpoint signaling. The molecular requirements for formation of the fibrous corona are not fully understood. Here, we show that the fibrous corona depends on the mitotic kinesin CENP-E and that poorly expanded fibrous coronas after CENP-E depletion are functionally compromised. This previously unrecognized role for CENP-E does not require its motor activity but instead is driven by farnesyl modification of its C-terminal kinetochore- and microtubule-binding domain. We show that in cells, CENP-E binds Spindly and recruits RZZ-S complexes to ectopic locations in a farnesyl-dependent manner. CENP-E is recruited to kinetochores following RZZ-S, and—while not required for RZZ-S oligomerization per se—promotes subsequent fibrous corona expansion. Our comparative genomics analyses suggest that the farnesylation motif in CENP-E orthologs emerged alongside the full RZZ-S module in an ancestral lineage close to the fungi–animal split (Obazoa), revealing potential conservation of the mechanisms for fibrous corona formation. Our results show that proper spindle assembly has a potentially conserved non-motor contribution from the kinesin CENP-E through stabilization of the fibrous corona meshwork during its formation.

## Teaser

Wu et al. show that the mitotic kinesin CENP-E promotes full expansion of the outer layer of the kinetochore known as the fibrous corona. This new function of CENP-E is independent of its motor activity. The findings expand the roles of CENP-E in spindle assembly and chromosome congression in mitosis.

## Introduction

The cell division cycle culminates in the segregation of duplicated sister chromatids to nascent daughter cells. Segregation is driven by the microtubule-based spindle apparatus (Pavin and Tolić, 2021). Microtubules connect to chromosomes via kinetochores, large protein complexes that assemble on centromeric chromatin and that contain microtubule-binding proteins (Cheeseman, 2014; Musacchio and Desai, 2017). To facilitate spindle assembly, kinetochores transiently expand in early mitosis by forming a voluminous protein meshwork known as the fibrous corona (Kops and Gassmann, 2020). The fibrous corona can make extensive connections to microtubule lattices (Kapoor et al., 2006; Rieder and Alexander, 1990; Sacristan et al., 2018), promotes chromosome orientation on the spindle (Magidson et al., 2015), is a microtubule nucleation platform (Mitchison and Kirschner, 1985; Ris and Witt, 1981; Witt et al., 1980; Wu et al., 2023), and strengthens spindle assembly checkpoint (SAC) signaling (Allan et al., 2020; Rodriguez-Rodriguez et al., 2018; Zhang et al., 2019).

The fibrous corona houses over two dozen proteins to perform its various roles in spindle assembly and chromosome segregation (Kops and Gassmann, 2020). It is formed by oligomerization of ROD/ZW10/ZWILCH-SPINDLY (RZZ-S) complexes (Mosalaganti et al., 2017; Pereira et al., 2018; Sacristan et al., 2018), which directly bind the dynein/dynactin microtubule motor complex (Chan et al., 2009; d’Amico et al., 2022; Gama et al., 2017; Griffis et al., 2007; Sacristan et al., 2018; Starr et al., 1998). Dynein/dynactin aids in microtubule capture and subsequent poleward transport of chromosomes along the microtubule lattice to facilitate eventual biorientation (Li et al., 2007; Savoian et al., 2000; Vorozhko et al., 2008; Yang et al., 2007). In addition, the LIC1 subunit of dynein promotes microtubule nucleation from the fibrous corona through interaction with the centrosomal protein pericentrin (Wu et al., 2023). Once kinetochores engage in end-on microtubule connections, dynein/dynactin drives the disassembly of the fibrous corona by “stripping” RZZ-S complexes and associated proteins from kinetochores (Basto et al., 2004; Famulski et al., 2011; Howell et al., 2001; Wojcik et al., 2001).

Other proteins of the fibrous corona include the SAC complex MAD1–MAD2–p31^comet^, the Cyclin B1–CDK1 kinase complex, the CLASP1/2 microtubule dynamics regulators, and the microtubule-binding protein centromere protein F (CENP-F; (Kops and Gassmann, 2020). The fibrous corona additionally harbors CENP-E (Cooke et al., 1997; Thrower et al., 1996; Yao et al., 1997), a kinesin-7 family microtubule motor that promotes correct orientation of microtubule stubs near the kinetochore, stable microtubule interactions, and congression of polar chromosomes (Kapoor et al., 2006; McEwen et al., 2001; Putkey et al., 2002; Schaar et al., 1997; Sikirzhytski et al., 2018; Wood et al., 1997). CENP-E is a dimer with amino-terminal kinesin domains and carboxy-terminal microtubule-binding domains, separated by a discontinuous ∼230-nm coiled-coil (Kim et al., 2008; Yen et al., 1992). The carboxy terminus of human CENP-E contains a CAAX box (with sequence Cys-Lys-Thr-Gln), which is a signal for farnesyl modification on the motif’s cysteine residue (Ashar et al., 2000).

CENP-E localizes to distinct locations on kinetochores. Besides a pool that localizes to fibrous coronas and is stripped by dynein/dynactin upon end-on microtubule binding (Howell et al., 2001), another pool of CENP-E, presumably bound to the core kinetochore, localizes regardless of whether kinetochores are bound to microtubules, and remains associated with kinetochores in metaphase cells (Weaver et al., 2003; Yen et al., 1991, 1992). While a direct interaction of CENP-E with BUBR1 likely contributes to the latter pool (Chan et al., 1998; Ciossani et al., 2018; Legal et al., 2020; Yao et al., 2000), the mechanism for CENP-E’s localization to the fibrous corona is not clear but appears to involve its farnesylation. Farnesyltransferase inhibitor treatment or mutation of the farnesyl-acceptor cysteine residue impacts CENP-E levels most prominently on prometaphase kinetochores and, in cell lines where fibrous coronas can be distinguished, prevents CENP-E from localizing to their crescent shapes (Ciossani et al., 2018; Holland et al., 2015; Schafer-Hales et al., 2007).

Fibrous corona formation in early mitosis is driven by oligomerization of RZZ-S complexes, a process that can be recapitulated by purified components in vitro (Pereira et al., 2018; Raisch et al., 2022; Sacristan et al., 2018). It is initiated by phosphorylation of the β-propeller domain of ROD by the kinetochore kinase MPS1 and involves conformational transitions in SPINDLY (Raisch et al., 2022; Rodriguez-Rodriguez et al., 2018; Sacristan et al., 2018). These events are likely to be mechanistically connected, as fibrous corona expansion no longer requires MPS1 activity when cells express a SPINDLY mutant mimicking its conformationally “active” state (Sacristan et al., 2018). While certainly required, whether these mechanisms are also sufficient for fibrous corona formation in cells is unknown. Here, we show that CENP-E has an important and possibly evolutionary conserved structural role in fibrous corona formation. Our findings expand the repertoire of CENP-E contributions to chromosome segregation and reveal new aspects of the molecular mechanisms driving full expansion of the fibrous corona meshwork in mitosis.

## Results

### Fibrous corona formation requires CENP-E

**Figure 1.**
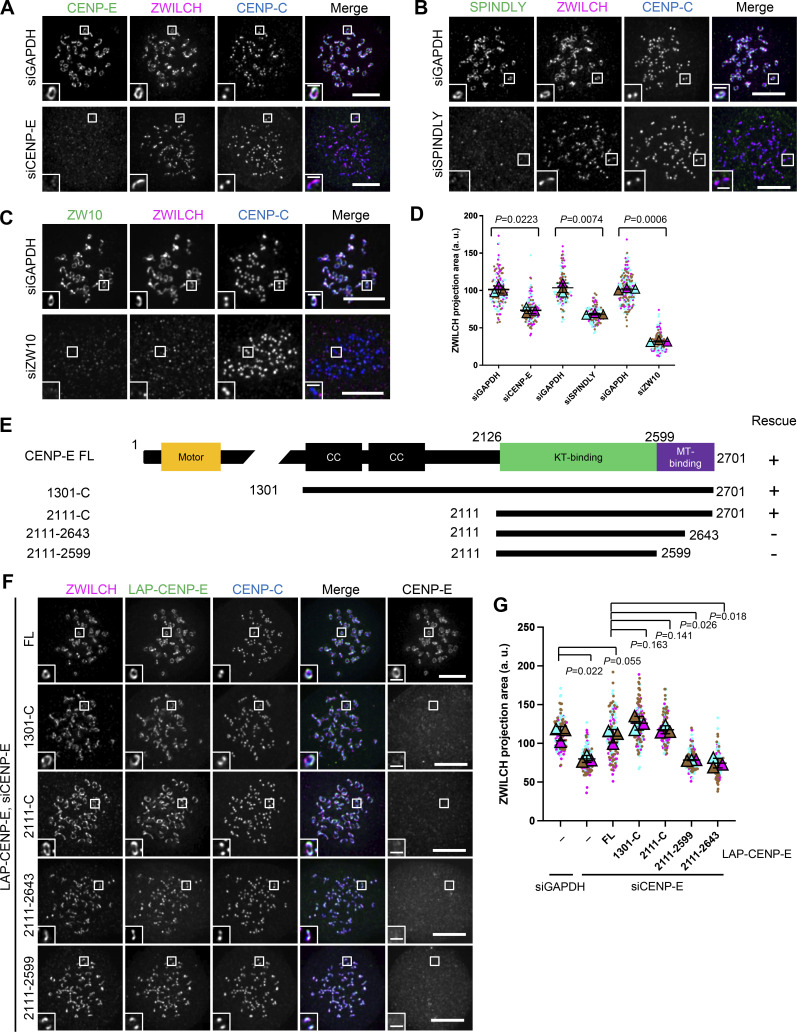
**Fibrous corona expansion requires CENP-E. (A)** Immunostaining of ZWILCH (magenta), CENP-E (green), and CENP-C (blue) in control and CENP-E–depleted RPE1 cells treated with nocodazole overnight. Bar, 5 μm; inset bar, 1 μm. **(B)** Immunostaining of ZWILCH (magenta), SPINDLY (green), and CENP-C (blue) in control and SPINDLY-depleted RPE1 cells treated with nocodazole overnight. Bar, 5 μm; inset bar, 1 μm. **(C)** Immunostaining of ZWILCH (magenta), ZW10 (green), and CENP-C (blue) in control and ZW10-depleted RPE1 cells treated with nocodazole overnight. Bar, 5 μm; inset bar, 1 μm. **(D)** Projection area of ZWILCH in control, CENP-E–depleted, SPINDLY-depleted, and ZW10-depleted RPE1 cells treated with nocodazole overnight. *n* > 120 pairs of kinetochores were quantified for each condition; two pairs of kinetochores per cell. Experiments were performed in triplicate and data from each trial was color-coded. Average value of each trial is given by a filled triangle of the corresponding color. Results are represented as mean ± SD; error bars represent SD. The P values represent a paired two-sample two-tailed *t* test. **(E)** Domain organization of CENP-E and the deletion mutants used to rescue fibrous corona. CC, coiled coil. Rescue of fibrous corona are indicated. **(F)** Immunostaining of ZWILCH (magenta), CENP-E (gray), and CENP-C (blue) in CENP-E–depleted RPE1 cells that overexpress the indicated LAP-tagged CENP-E mutant after nocodazole treatment overnight. Bar, 5 μm; inset bar, 1 μm. Rabbit polyclonal CENP-E antibody recognizes human CENP-E 955–1571. LAP: localization and affinity purification. **(G)** Projection area of ZWILCH in control and CENP-E–depleted RPE1 cells and CENP-E–depleted RPE1 cells that inducibly overexpress the indicated LAP-tagged CENP-E mutant with nocodazole treatment overnight. *n* > 120 pairs of kinetochores were quantified for each condition; two pairs of kinetochores per cell. Experiments were performed in triplicate and data from each trial was color-coded. Average value of each trial is given by a filled triangle of the corresponding color. Results are represented as mean ± SD; error bars represent SD. The P values represent a paired two-sample two-tailed *t* test.

**Figure S1.**
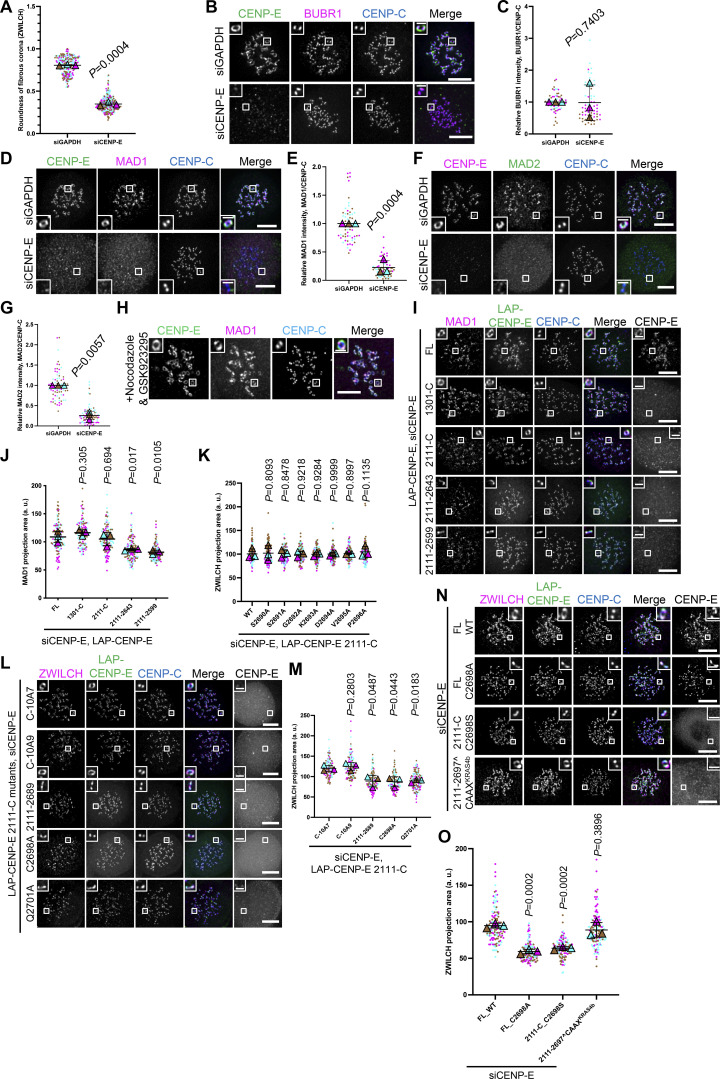
**Fibrous corona formation requires C-terminal farnesylation of CENP-E. (A)** The roundness of the shape of fibrous corona in control and CENP-E–knockdown RPE1 cells treated with nocodazole overnight. *n* > 240 pairs of kinetochores were quantified for each condition; four pairs of kinetochores per cell. Experiments were performed in triplicate and data from each trial was color-coded. Average value of each trial is given by a filled triangle of the corresponding color. Results are represented as mean ± SD; error bars represent SD. The P value represents a paired two-sample two-tailed *t* test. **(B)** Immunostaining of BUBR1 (magenta), CENP-E (green), and CENP-C (blue) in control and CENP-E–knockdown RPE1 cells treated with nocodazole overnight. Bar, 5 μm; inset bar, 1 μm. **(C)** Relative BUBR1 intensity at the kinetochore compared with CENP-C in control and CENP-E–knockdown RPE1 cells treated with nocodazole overnight. *n* > 60 cells were quantified for each condition. Experiments were performed in triplicate and data from each trial was color-coded. Average value of each trial is given by a filled triangle of the corresponding color. Results are represented as mean ± SD; error bars represent SD. The P value represents a paired two-sample two-tailed *t* test. **(D)** Immunostaining of MAD1 (magenta), CENP-E (green), and CENP-C (blue) in control and CENP-E–depleted RPE1 cells treated with nocodazole overnight. Bar, 5 μm; inset bar, 1 μm. **(E)** Relative intensity of MAD1 on the kinetochore in control and CENP-E–depleted RPE1 cells with nocodazole treatment overnight. *n* > 60 cells were quantified for each condition. Experiments were performed in triplicate and data from each trial was color-coded. Average value of each trial is given by a filled triangle of the corresponding color. Results are represented as mean ± SD; error bars represent SD. The P value represents a paired two-sample two-tailed *t* test. **(F)** Immunostaining of CENP-E (magenta), MAD2 (green), and CENP-C (blue) in control and CENP-E–depleted RPE1 cells treated with nocodazole overnight. Bar, 5 μm; inset bar, 1 μm. **(G)** Relative intensity of MAD2 on the kinetochore in control and CENP-E–depleted RPE1 cells with nocodazole treatment overnight. *n* > 60 cells were quantified for each condition. Experiments were performed in triplicate and data from each trial was color-coded. Average value of each trial is given by a filled triangle of the corresponding color. Results are represented as mean ± SD; error bars represent SD. The P values represent a paired two-sample two-tailed *t* test. **(H)** Immunostaining of CENP-E (magenta), MAD1 (green), and CENP-C (blue) in RPE1 cells cotreated with nocodazole and GSK923295 (inhibitor of CENP-E motor activity) overnight. Bar, 5 μm; inset bar, 1 μm. **(I)** Immunostaining of MAD1 (magenta), CENP-E (gray), and CENP-C (blue) in CENP-E–depleted RPE1 cells that overexpress the indicated LAP-tagged CENP-E mutant after nocodazole treatment overnight. Bar, 5 μm; inset bar, 1 μm. Rabbit polyclonal CENP-E antibody recognizes human CENP-E 955–1571. **(J)** Projection area of MAD1 in CENP-E–depleted RPE1 cells that inducibly overexpress the indicated LAP-tagged CENP-E mutant with nocodazole treatment overnight. *n* > 120 pairs of kinetochores were quantified for each condition; two pairs of kinetochores per cell. Experiments were performed in triplicate and data from each trial was color-coded. Average value of each trial is given by a filled triangle of the corresponding color. Results are represented as mean ± SD; error bars represent SD. The P values represent a paired two-sample two-tailed *t* test. **(K)** Projection area of ZWILCH in CENP-E–knockdown HeLa cells that overexpress the indicated LAP-tagged CENP-E mutant after 8 h nocodazole treatment. *n* > 120 pairs of kinetochores were quantified for each condition; two pairs of kinetochores per cell. Experiments were performed in triplicate and data from each trial was color-coded. Average value of each trial is given by a filled triangle of the corresponding color. Results are represented as mean ± SD; error bars represent SD. The P values represent a paired two-sample two-tailed *t* test. **(L)** Immunostaining of ZWILCH (magenta), CENP-E (gray), and CENP-C (blue) in CENP-E–knockdown RPE1 cells that overexpress the indicated LAP-tagged CENP-E mutant after nocodazole treatment overnight. Bar, 5 μm; inset bar, 1 μm. Rabbit polyclonal CENP-E antibody recognizes human CENP-E 955–1571. **(M)** Projection area of ZWILCH in CENP-E–knockdown RPE1 cells that inducibly overexpress the indicated LAP-tagged CENP-E mutant with nocodazole treatment overnight. *n* > 120 pairs of kinetochores were quantified for each condition; two pairs of kinetochores per cell. Experiments were performed in triplicate and data from each trial was color-coded. Average value of each trial is given by a filled triangle of the corresponding color. Results are represented as mean ± SD; error bars represent SD. The P values represent a paired two-sample two-tailed *t* test. **(N)** Immunostaining of ZWILCH (magenta), CENP-E (gray), and CENP-C (blue) in CENP-E–depleted HeLa cells that overexpress the indicated CENP-E mutant after 6 h nocodazole treatment. Bar, 5 μm; inset bar, 1 μm. **(O)** Projection area of ZWILCH in CENP-E–depleted HeLa cells that overexpress the indicated CENP-E mutant after 6 h nocodazole treatment. *n* > 120 pairs of kinetochores were quantified for each condition; two pairs of kinetochores per cell. Experiments were performed in triplicate and data from each trial was color-coded. Average value of each trial is given by a filled triangle of the corresponding color. Results are represented as mean ± SD; error bars represent SD. The P values represent a paired two-sample two-tailed *t* test.

Being a constituent of the fibrous corona (Cooke et al., 1997; Thrower et al., 1996; Yao et al., 1997), we wondered whether CENP-E contributes to fibrous corona formation. The area of the fibrous corona in nocodazole-treated RPE1 cells, marked by the RZZ component ZWILCH (Mosalaganti et al., 2017; Pereira et al., 2018), was substantially reduced when CENP-E was depleted by RNAi (Fig. 1, A and D). Depletion of CENP-E affected fibrous corona size and shape to a similar extent as loss of SPINDLY (Fig. 1, A–D; and Fig. S1 A; Pereira et al., 2018; Sacristan et al., 2018). CENP-E depletion did not affect local levels of its outer kinetochore receptor BUBR1 (Fig. S1, B and C) but strongly reduced the fibrous corona-localized SAC complex MAD1–MAD2 (Fig. S1, D–G).

To determine how CENP-E contributes to fibrous corona formation, we next performed RNAi complementation with a series of LAP-tagged CENP-E mutants (Fig. 1 E). This revealed that the motor domain of CENP-E was dispensable for fibrous corona formation (Fig. 1, F and G). Consistently, the fibrous corona was unaffected by treatment with GSK923295, a small molecular inhibitor of CENP-E motor activity (Fig. S1 H; Qian et al., 2010). Instead, a fragment of CENP-E encompassing amino acids 2,111–2,701 (hereafter named 2111-C), including the kinetochore-binding (KT-binding) and microtubule-binding (MT-binding) domains, was sufficient to rescue fibrous corona formation in the absence of endogenous CENP-E. This depended on the C-terminal 58 amino acids within the MT-binding domain (Fig. 1, F and G; and Fig. S1, I and J).

### Fibrous corona formation requires C-terminal farnesylation of CENP-E

**Figure 2.**
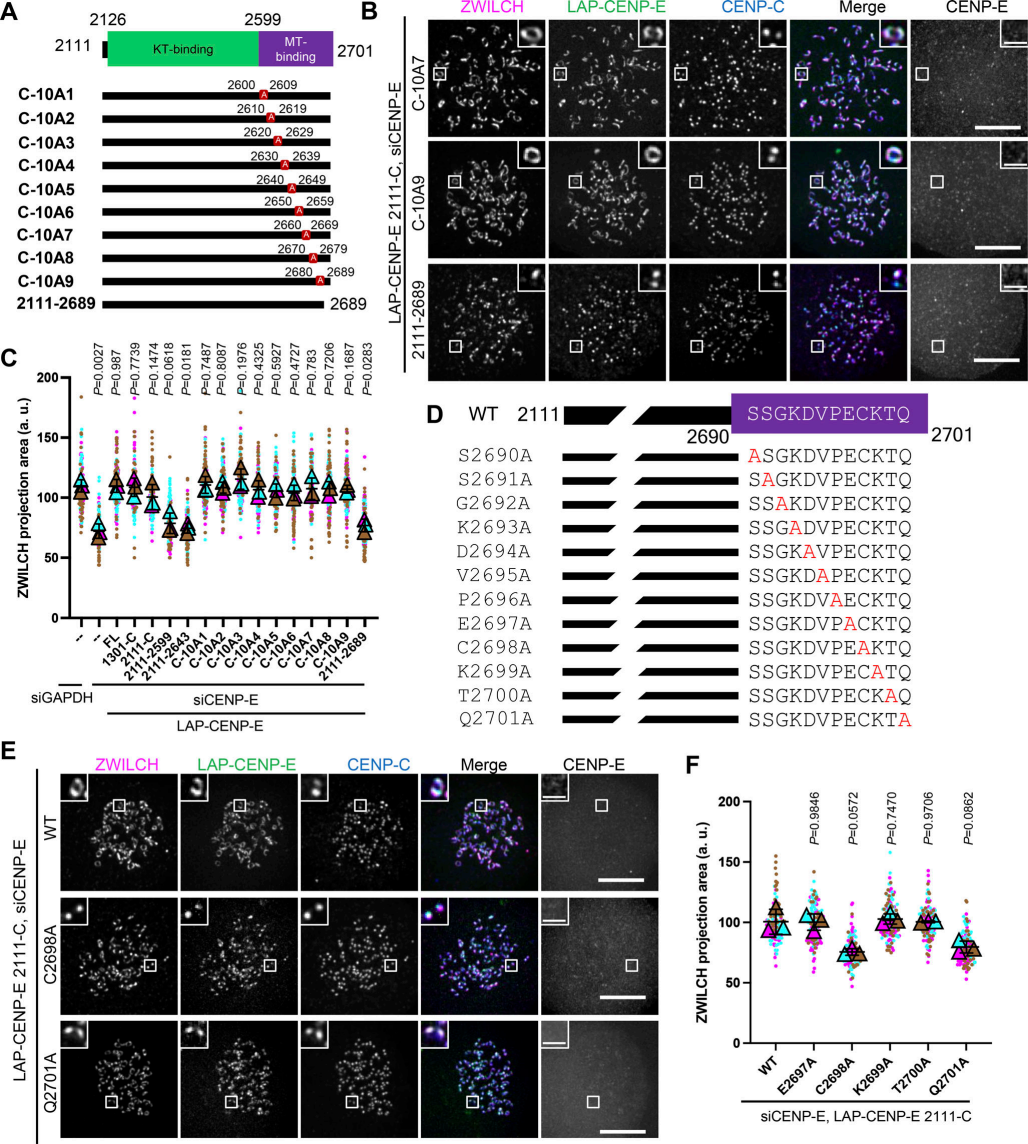
**Fibrous corona formation requires C-terminal farnesylation of CENP-E. (A)** Domain organization of CENP-E 2111-C and various respective mutants. 2111-C fragment of CENP-E with different stretches of 10 amino acids were substituted for alanine residues or was deleted of the C-terminal 12 amino acids. **(B)** Immunostaining of ZWILCH (magenta), CENP-E (gray), and CENPC (blue) in CENP-E–knockdown HeLa cells that overexpress the indicated CENP-E mutant after 8 h nocodazole treatment. Bar, 5 μm; inset bar, 1 μm. Rabbit polyclonal CENP-E antibody recognizes human CENP-E 955–1571. **(C)** Projection area of ZWILCH in control and CENP-E–knockdown HeLa cells and CENP-E–knockdown HeLa cells that inducibly overexpress the indicated LAP-tagged CENP-E mutant after 8 h nocodazole treatment. *n* > 120 pairs of kinetochores were quantified for each condition; two pairs of kinetochores per cell. Experiments were performed in triplicate and data from each trial was color-coded. Average value of each trial is given by a filled triangle of the corresponding color. Results are represented as mean ± SD; error bars represent SD. The P values represent a paired two-sample two-tailed *t* test. **(D)** Domain organization of CENP-E C-terminal and the indicated point-mutated mutants used to rescue fibrous corona. **(E)** Immunostaining of ZWILCH (magenta), CENP-E (gray), and CENP-C (blue) in CENP-E–knockdown HeLa cells that overexpress the indicated LAP-tagged CENP-E mutant after 8 h nocodazole treatment. Bar, 5 μm; inset bar, 1 μm. Rabbit polyclonal CENP-E antibody recognizes human CENP-E 955–1571. **(F)** Projection area of ZWILCH in CENP-E–knockdown HeLa cells that overexpress the indicated LAP-tagged CENP-E mutant after 8 h nocodazole treatment. *n* > 120 pairs of kinetochores were quantified for each condition; two pairs of kinetochores per cell. Experiments were performed in triplicate and data from each trial was color-coded. Average value of each trial is given by a filled triangle of the corresponding color. Results are represented as mean ± SD; error bars represent SD. The P values represent a paired two-sample two-tailed *t* test.

Having found that the C-terminal-most part of CENP-E (2111-C) was fully sufficient to restore fibrous corona formation after CENP-E loss, we next attempted to further narrow down the essential region. Expression in HeLa cells of a series of MT-binding domain mutants of CENP-E 2111-C (Fig. 2 A) revealed that the C-terminal 12 amino acids were essential for fibrous corona formation (Fig. 2, B and C). This sequence contains a CAAX box, which is a farnesylation motif (Ashar et al., 2000). We have previously shown that cells treated with the farnesylation inhibitor lonafarnib do not expand fibrous coronas, a phenotype also seen in cells expressing a SPINDLY mutant that cannot be farnesylated (Sacristan et al., 2018). Mutation of C2698, the farnesyl acceptor residues of CENP-E’s CAAX box, abrogated the ability of 2111-C and full-length (FL) CENP-E to support fibrous corona expansion in HeLa and RPE1 cells (Fig. 2, D–F; and Fig. S1, K–O). Consistently, mutation of the only other residue in the CAAX box important for the farnesylation signal, Q2701, but not any of the other C-terminal 12 amino acids, prevented fibrous corona formation (Fig. 2, D–F; and Fig. S1, K–M). Moreover, the CAAX box of Kirsten rat sarcoma viral oncogene homolog (KRAS) isoform 4b (KRAS4b, sequence Cys-Ile-Ile-Met; Dharmaiah et al., 2016) could functionally replace the CAAX box of CENP-E (Fig. S1, N and O). We conclude that farnesylation of CENP-E is required for fibrous corona formation.

### Farnesylated CENP-E is essential for kinetochore-derived microtubule nucleation and chromosome congression

**Figure 3.**
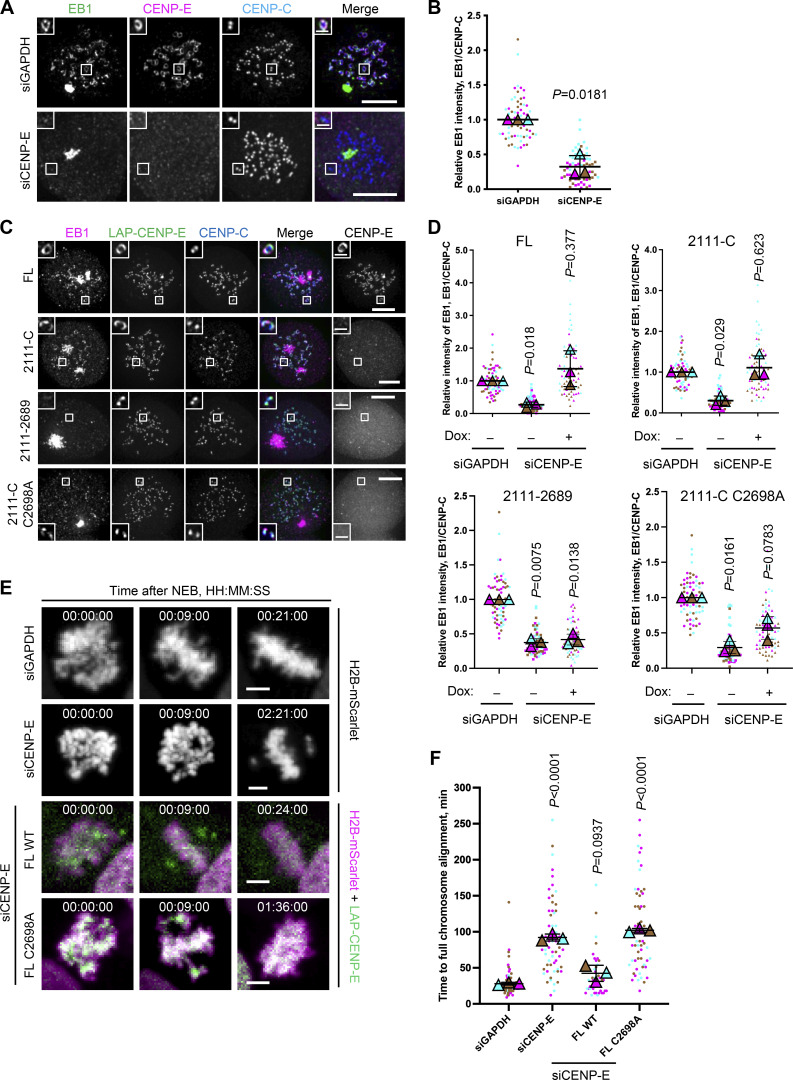
**Farnesylated CENP-E is essential for kinetochore-derived microtubule nucleation. (A)** Immunostaining of EB1 (green), CENP-E (magenta), and CENP-C (blue) in control and CENP-E–knockdown RPE1 cells after recovery of 3 min from nocodazole washout. Bar, 5 μm; inset bar, 1 μm. **(B)** Relative EB1 intensity at the kinetochore compared with CENP-C in control and CENP-E–knockdown RPE1 cells after recovery of 3 min from nocodazole washout. *n* > 60 cells were quantified for each condition. Experiments were performed in triplicate and data from each trial was color-coded. Average value of each trial is given by a filled triangle of the corresponding color. Results are represented as mean ± SD; error bars represent SD. The P values represent a paired two-sample two-tailed *t* test. **(C)** Immunostaining of EB1 (magenta), CENP-E (gray), and CENP-C (blue) in CENP-E–knockdown RPE1 cells that overexpress the indicated CENP-E mutant after recovery of 2.5–3 min from nocodazole washout. Bar, 5 μm; inset bar, 1 μm. Rabbit polyclonal CENP-E antibody recognizes human CENP-E 955–1571. **(D)** Relative EB1 intensity at the kinetochore compared with CENP-C in control and CENP-E–knockdown RPE1 cells that inducibly overexpress the indicated CENP-E mutant after recovery of 2.5–3 min from nocodazole washout. *n* > 60 cells were quantified for each condition. Experiments were performed in triplicate and data from each trial was color-coded. Average value of each trial is given by a filled triangle of the corresponding color. Results are represented as mean ± SD; error bars represent SD. The P values represent a paired two-sample two-tailed *t* test. **(E)** Stills from live imaging of control and CENP-E–knockdown HeLa cells and CENP-E–knockdown HeLa cells that inducibly overexpress the indicated LAP-tagged CENP-E mutant. The cells used in this experiment overexpress H2B-mScarlet. Bar, 5 μm. **(F)** Time taken from nuclear envelope breakdown to chromosome alignment in control and CENP-E–knockdown HeLa cells and CENP-E–knockdown HeLa cells that inducibly overexpress the indicated LAP-tagged CENP-E mutant. *n* = 44–80 cells were quantified for each condition. Experiments were performed in triplicate and data from each trial was color-coded. Average value of each trial is given by a filled triangle of the corresponding color. Results are represented as mean ± SD; error bars represent SD. The P values represent a paired two-sample two-tailed *t* test.

**Figure S2.**
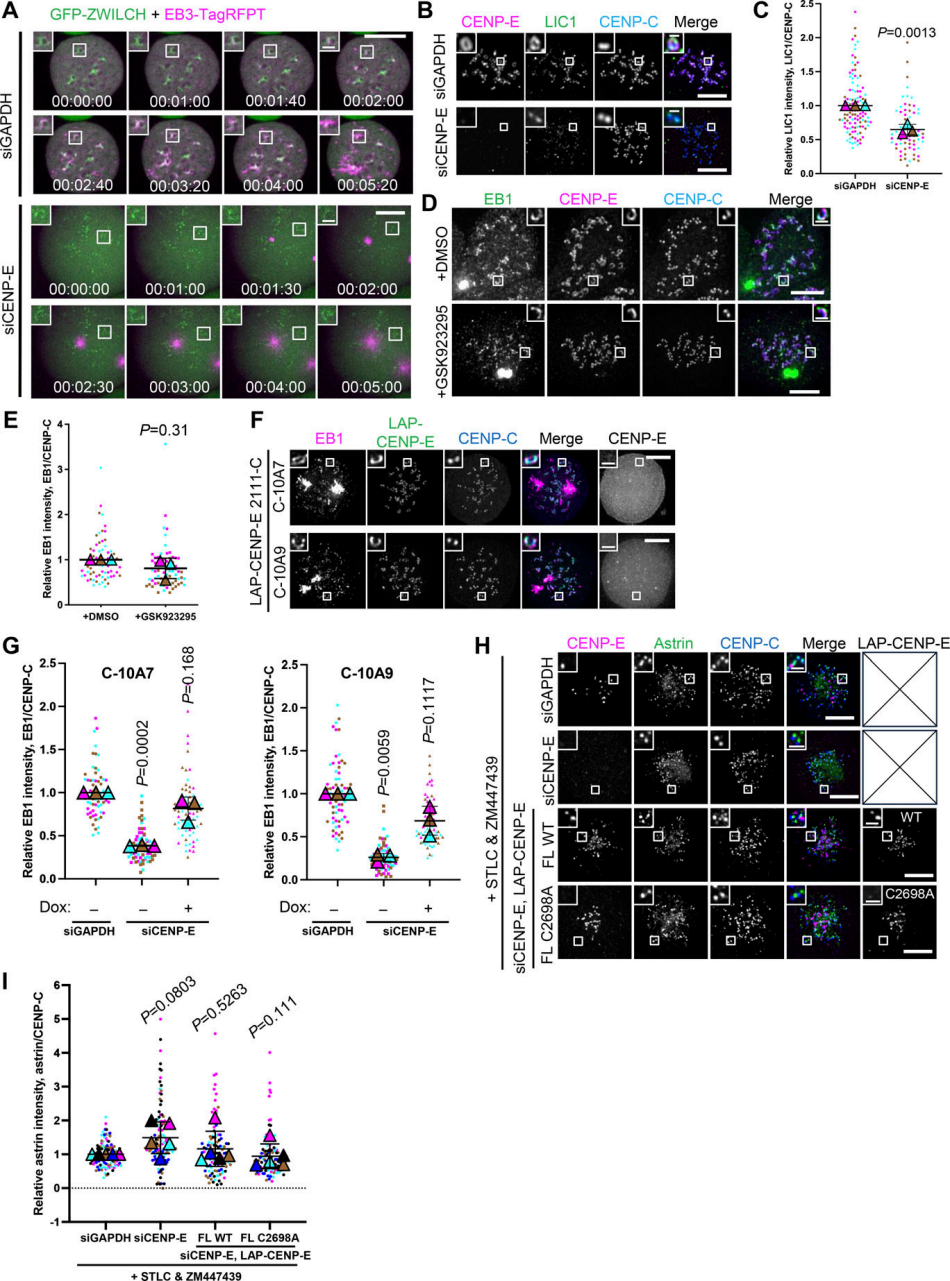
**Farnesylated CENP-E is essential for kinetochore-derived microtubule nucleation. (A)** Stills from live imaging of control and CENP-E–knockdown RPE1 cells that coexpress GFP-ZWILCH and EB3-TagRFPT after nocodazole washout. Bar, 5 μm; inset bar, 1 μm. **(B)** Immunostaining of CENP-E (magenta), LIC1 (green), and CENP-C (blue) in control and CENP-E–depleted RPE1 cells treated with nocodazole overnight. Bar, 5 μm; inset bar, 0.5 μm. **(C)** Relative LIC1 intensity at the kinetochore compared with CENP-C in control and CENP-E–knockdown RPE1 cells treated with nocodazole overnight. *n* > 60 cells were quantified for each condition. Experiments were performed in triplicate and data from each trial was color-coded. Average value of each trial is given by a filled triangle of the corresponding color. Results are represented as mean ± SD; error bars represent SD. The P values represent a paired two-sample two-tailed *t* test. **(D)** Immunostaining of EB1 (green), CENP-E (magenta), and CENP-C (blue) in DMSO and GSK923295-treated RPE1 cells after recovery of 2.5 min from nocodazole washout. Bar, 5 μm; inset bar, 1 μm. **(E)** Relative EB1 intensity at the kinetochore compared to CENP-C in DMSO and GSK923295-treated RPE1 cells after recovery of 2.5 min from nocodazole washout. *n* > 60 cells were quantified for each condition. Experiments were performed in triplicate and data from each trial was color-coded. Average value of each trial is given by a filled triangle of the corresponding color. Results are represented as mean ± SD; error bars represent SD. The P values represent a paired two-sample two-tailed *t* test. **(F)** Immunostaining of EB1 (magenta), CENP-E (gray), and CENP-C (blue) in CENP-E–knockdown RPE1 cells that overexpress the indicated CENP-E mutant after recovery of 3 min from nocodazole washout. Bar, 5 μm; inset bar, 1 μm. Rabbit polyclonal CENP-E antibody recognizes human CENP-E 955–1571. **(G)** Relative EB1 intensity at the kinetochore compared to CENP-C in control and CENP-E–knockdown RPE1 cells that inducibly overexpress the indicated CENP-E mutant after recovery of 3 min from nocodazole washout. *n* > 60 cells were quantified for each condition. Experiments were performed in triplicate and data from each trial was color-coded. Average value of each trial is given by a filled triangle of the corresponding color. Results are represented as mean ± SD; error bars represent SD. The P values represent a paired two-sample two-tailed *t* test. **(H)** Immunostaining of CENP-E (magenta), astrin (green), and CENP-C (blue) in control and CENP-E–depleted HeLa cells and CENP-E–depleted HeLa cells that overexpress the indicated LAP-tagged CENP-E mutant (gray) after 6 h treatment of STLC and ZM447439. Bar, 5 μm; inset bar, 1 μm. **(I)** Relative astrin intensity at the kinetochore compared with CENP-C in control and CENP-E–depleted HeLa cells and CENP-–-depleted HeLa cells that overexpress the indicated LAP-tagged CENP-E mutant after 6 h treatment of STLC and ZM447439. *n* > 100 cells were quantified for each condition. Experiments were performed in quintuplicate and data from each trial was color-coded. Average value of each trial is given by a filled triangle of the corresponding color. Results are represented as mean ± SD; error bars represent SD. The P values represent a paired two-sample two-tailed *t* test.

Our prior work showed that fibrous coronas are the site of kinetochore-dependent microtubule nucleation, which aids spindle assembly and chromosome congression (Wu et al., 2023). To examine whether the contribution of CENP-E to fibrous corona expansion impacted fibrous corona function, we next assessed whether kinetochore-derived microtubule nucleation depends on CENP-E. To this end, we washed out nocodazole from RPE1 cells that had been treated with nocodazole overnight and assessed microtubule regrowth from fibrous coronas. In contrast to control cells, which had abundant microtubules newly nucleated on kinetochores 3 min after washout of nocodazole, cells depleted of CENP-E showed virtually no microtubule nucleation on kinetochores (Fig. 3, A and B; and Fig. S2 A). Consistent with our prior observation that fibrous corona–derived microtubule nucleation requires the LIC1 subunit of dynein (Wu et al., 2023), depletion of CENP-E substantially removed LIC1 from kinetochores (Fig. S2, B and C). Inhibition of CENP-E motor activity by treatment with GSK923295 did not prevent kinetochore-derived microtubule nucleation (Fig. S2, D and E), and, consistently, neither did expression of the 2111-C fragment, which lacks the motor domain (Fig. 3, C and D). These results indicate that CENP-E promotes microtubule nucleation at kinetochores through its non-motor role in fibrous corona expansion. In support of this, the mutants of CENP-E lacking the CAAX box (2,111–2,689) or in which the farnesyl-acceptor cysteine is substituted (C2698A) failed to restore microtubule nucleation (Fig. 3, C and D; and Fig. S2, F and G). As expected (Holland et al., 2015; Wu et al., 2023), replacing endogenous CENP-E with CENP-E^C2698A^ delayed chromosome congression (Fig. 3 E) without substantially compromising the ability of kinetochores to form stable end-on attachments (Fig. S2, H and I). Together, our findings show that CENP-E is essential for fibrous corona expansion and function in spindle assembly.

### A farnesylated CENP-E fragment can recruit the RZZ-S complex in interphase cells

**Figure 4.**
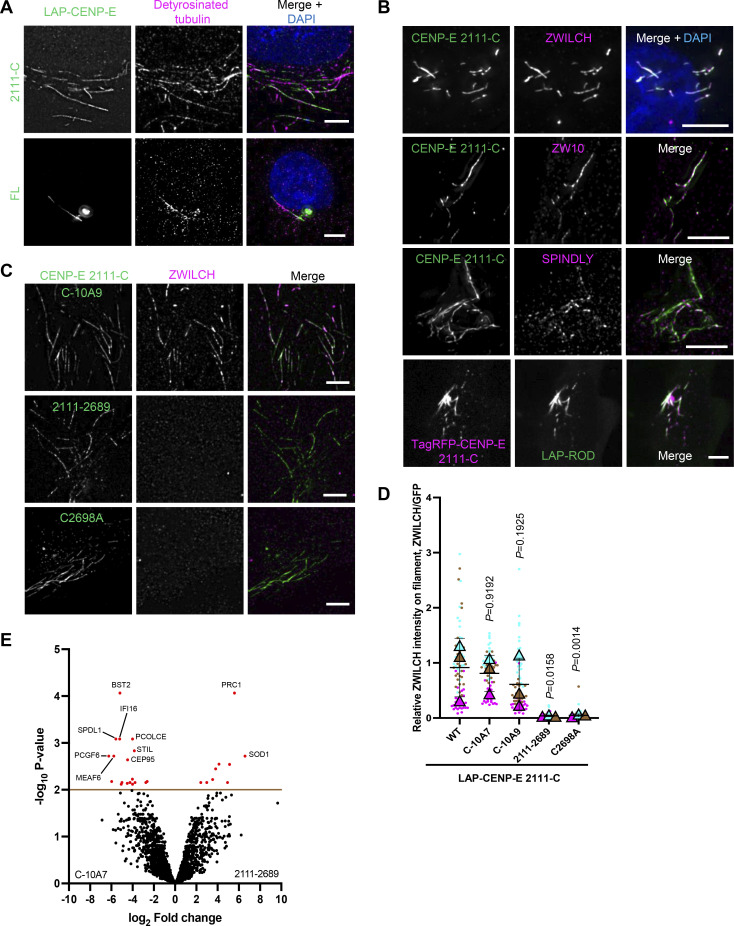
**A farnesylated CENP-E fragment can recruit the RZZ-S complex in interphase cells. (A)** Immunostaining of detyrosinated tubulin (magenta) in RPE1 cells that overexpress LAP-CENP-E 2111-C/FL. Bar, 5 μm. **(B)** Immunostaining of ZWILCH/ZW10/SPINDLY (magenta) in HeLa cells that overexpress LAP-CENP-E 2111-C (top three panels) and HeLa cells that coexpress TagRFP-CENP-E 2111-C and LAP-ROD. Bar, 5 μm. **(C)** Immunostaining of ZWILCH (magenta) in RPE1 cells that overexpress the indicated LAP-tagged CENP-E mutants. Bar, 5 μm. **(D)** Relative intensity of ZWILCH on the microtubule filaments bound with indicated CENP-E mutants. *n* = 150 filaments were quantified for each condition; two filaments per cell. Experiments were performed in triplicate and data from each trial was color-coded. Average value of each trial is given by a filled triangle of the corresponding color. Results are represented as mean ± SD; error bars represent SD. The P values represent a paired two-sample two-tailed *t* test. **(E)** Volcano plot shows top enriched proteins bound to the streptavidin beads incubation with extracts of HeLa cells expressing LAP-TurboID-CENP-E 2111-C C-10A7 (left) or LAP-TurboID-CENP-E 2,111–2,689 (right).

**Figure S3.**
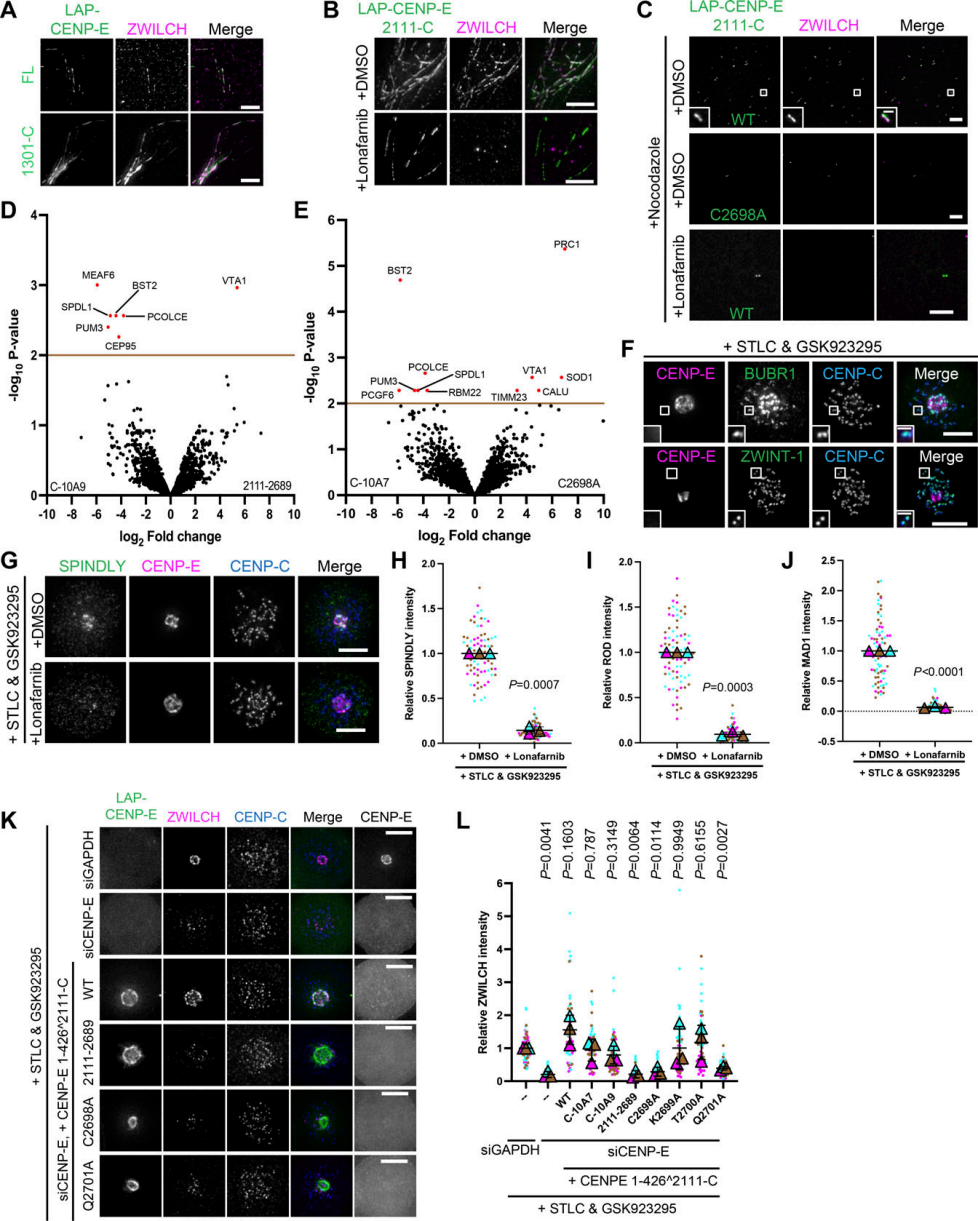
**Farnesylation promotes interaction of CENP-E with fibrous corona proteins. (A)** Immunostaining of ZWILCH (magenta) in RPE cells that overexpress LAP-CENP-E FL or LAP-CENP-E 1301-C. Bar, 5 μm. **(B)** Immunostaining of ZWILCH (magenta) in control and 5uM lonafarnib-treated HeLa cells that overexpress LAP-CENP-E 2111-C. Bar, 5 μm. **(C)** Immunostaining of ZWILCH (magenta) in DMSO/lonafarnib-treated RPE cells that overexpress LAP-CENP-E 2111-C and LAP-CENP-E 2111-C C2698A in the presence of nocodazole treatment. Bar, 5 μm; inset bar, 1 μm. **(D)** Volcano plot shows top enriched proteins bound to the streptavidin beads incubation with extracts of HeLa cells expressing LAP-TurboID-CENP-E 2111-C C-10A9 (left) or LAP-TurboID-CENP-E 2111-2689 (right). **(E)** Volcano plot shows top enriched proteins bound to the streptavidin beads incubation with extracts of HeLa cells expressing LAP-TurboID-CENP-E 2111-C C-10A7 (left) or LAP-TurboID-CENP-E 2111-C C2698A (right). **(F)** Immunostaining of BUBR1 (green)/ZWINT-1 (green), CENP-E (magenta), and CENP-C (blue) in RPE1 cells treated with STLC and GSK923295 overnight. Bar, 5 μm; inset bar, 1 μm. **(G)** Immunostaining of SPINDLY (green), CENP-E (magenta), and CENP-C (blue) in RPE1 cells treated with STLC and GSK923295 overnight in the presence of DMSO or 5 μM lonafarnib. Bar, 5 μm. **(H)** Relative intensity of SPINDLY at the spindle pole in RPE1 cells treated with STLC and GSK923295 overnight in the presence of DMSO or 5 μM lonafarnib. *n* > 60 cells were quantified for each condition. Experiments were performed in triplicate and data from each trial was color-coded. Average value of each trial is given by a filled triangle of the corresponding color. Results are represented as mean ± SD; error bars represent SD. The P values represent a paired two-sample two-tailed *t* test. **(I)** Relative intensity of ROD at the spindle pole in RPE1 cells treated with STLC and GSK923295 overnight in the presence of DMSO or 5 μM lonafarnib. *n* > 60 cells were quantified for each condition. Experiments were performed in triplicate and data from each trial was color-coded. Average value of each trial is given by a filled triangle of the corresponding color. Results are represented as mean ± SD; error bars represent SD. The P values represent a paired two-sample two-tailed *t* test. **(J)** Relative intensity of MAD1 at the spindle pole in RPE1 cells treated with STLC and GSK923295 overnight in the presence of DMSO or 5 μM lonafarnib. *n* > 60 cells were quantified for each condition. Experiments were performed in triplicate and data from each trial was color-coded. Average value of each trial is given by a filled triangle of the corresponding color. Results are represented as mean ± SD; error bars represent SD. The P values represent a paired two-sample two-tailed *t* test. **(K)** Immunostaining of ZWILCH (magenta), CENP-E (gray), and CENP-C (blue) in control and CENP-E–depleted HeLa cells and CENP-E–depleted HeLa cells that overexpress the indicated LAP-tagged CENP-E mutant after STLC and GSK923295 treatment overnight. Bar, 5 μm. LAP-tagged CENP-E mutants were created by fusing the N-terminal 1–426 (comprising the motor domain) to the C-terminal (KT-binding domain and MT-binding domain). Rabbit polyclonal CENP-E antibody recognizes human CENP-E 955–1571. **(L)** Relative intensity of ZWILCH at the spindle pole in control and CENP-E–depleted HeLa cells and CENP-E–depleted HeLa cells that inducibly overexpress the indicated LAP-tagged CENP-E mutant after STLC and GSK923295 treatment overnight. *n* > 60 cells were quantified for each condition. Experiments were performed in triplicate and data from each trial was color-coded. Average value of each trial is given by a filled triangle of the corresponding color. Results are represented as mean ± SD; error bars represent SD. The P values represent a paired two-sample two-tailed *t* test.

During our experiments, we noticed that the 2111-C mutant of CENP-E and occasionally also longer mutants, which all include a microtubule-binding domain, could be found to colocalize with stable microtubules in interphase cells (Fig. 4 A). Surprisingly, we found that RZZ-S complex proteins localized to these 2111-C–covered microtubules (Fig. 4 B and Fig. S3 A). Recruitment of RZZ-S (marked by ZWILCH) to these microtubules depended on farnesylation of the 2111-C fragment (Fig. 4, C and D; and Fig. S3 B). Cells treated with nocodazole to deplete microtubules still retained small clusters of 2111-C colocalizing with ZWILCH, in a manner that depended on 2111-C farnesylation (Fig. S3 C). We thus conclude that farnesylated 2111-C is sufficient to recruit RZZ-S to ectopic sites in interphase cells. To further explore the mechanisms by which RZZ-S is recruited to ectopic 2111-C locations, we performed proximity biotinylation proteomics on interphase HeLa cells expressing 2111-C mutants tagged with TurboID at their amino-termini (to not interfere with carboxy-terminal farnesylation; Branon et al., 2018). Compared to the biotinylated fractions from cells expressing unfarnesylated 2111-C mutants (2,111–2,689, C2698A), those from cells expressing farnesylated 2111-C mutants (C-10A7, C-10A9) were consistently enriched for SPINDLY (SPDL1; Fig. 4 E and Fig. S3, D and E). Farnesylated 2111-C might thus, directly or indirectly, recruit RZZ-S complexes via SPINDLY.

### Farnesylation promotes interaction of endogenous CENP-E with fibrous corona proteins in mitosis

**Figure 5.**
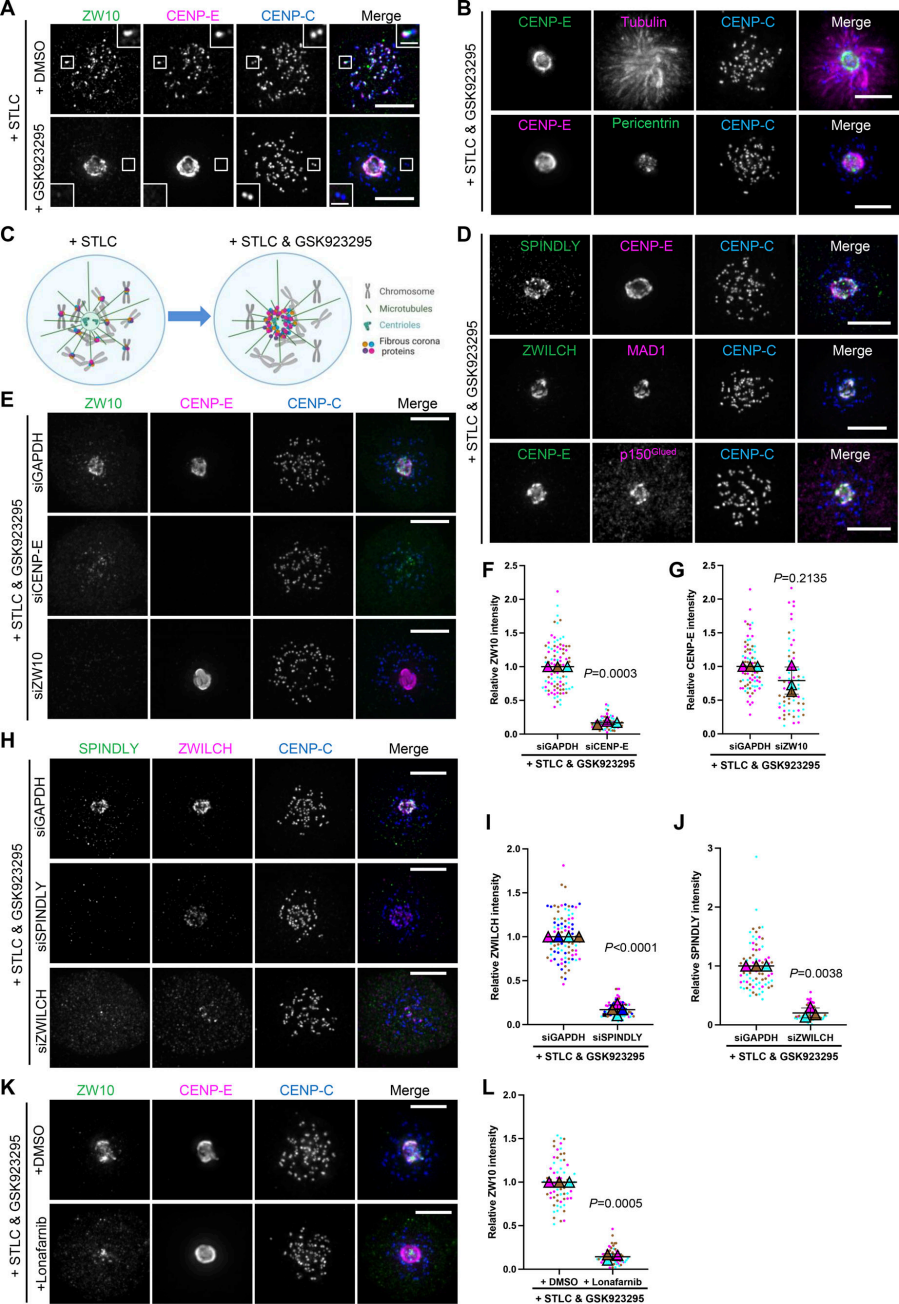
**Farnesylation promotes interaction of endogenous CENP-E with fibrous corona proteins in mitosis. (A)** Immunostaining of ZW10 (green), CENP-E (magenta), and CENP-C (blue) in STLC-treated RPE1 cells cotreated with DMSO or GSK923295 overnight. Bar, 5 μm; inset bar, 1 μm. **(B)** Immunostaining of CENP-E (green)/pericentrin (green), tubulin (magenta)/CENP-E (magenta), and CENP-C (blue) in RPE1 cells treated with STLC and GSK923295 overnight. Bar, 5 μm. **(C)** Cartoon showing the effects on fibrous corona proteins localization of indicated drugs treatment. **(D)** Immunostaining of SPINDLY (green)/ZWILCH (green)/CENP-E (green), CENP-E (magenta)/MAD1 (magenta)/p150^Glued^ (magenta), and CENP-C (blue) in RPE1 cells treated with STLC and GSK923295 overnight. Bar, 5 μm. **(E)** Immunostaining of ZW10 (green), CENP-E (magenta), and CENP-C (blue) in control, CENP-E–depleted, and ZW10-depleted RPE1 cells treated with STLC and GSK923295 overnight. Bar, 5 μm. **(F)** Relative intensity of ZW10 at the spindle pole in control and CENP-E–depleted RPE1 cells treated with STLC and GSK923295 overnight. *n* > 60 cells were quantified for each condition. Experiments were performed in triplicate and data from each trial was color-coded. Average value of each trial is given by a filled triangle of the corresponding color. Results are represented as mean ± SD; error bars represent SD. The P values represent a paired two-sample two-tailed *t* test. **(G)** Relative intensity of CENP-E at the spindle pole in control and ZW10-depleted RPE1 cells treated with STLC and GSK923295 overnight. *n* > 60 cells were quantified for each condition. Experiments were performed in triplicate and data from each trial were color-coded. Average value of each trial is given by a filled triangle of the corresponding color. Results are represented as mean ± SD; error bars represent SD. The P values represent a paired two-sample two-tailed *t* test. **(H)** Immunostaining of SPINDLY (green), ZWILCH (magenta), and CENP-C (blue) in control, SPINDLY-depleted, and ZWILCH-depleted RPE1 cells treated with STLC and GSK923295 overnight. Bar, 5 μm. **(I)** Relative intensity of ZWILCH at the spindle pole in control and SPINDLY-depleted RPE1 cells treated with STLC and GSK923295 overnight. *n* > 80 cells were quantified for each condition. Experiments were performed in quadruplicate and data from each trial was color-coded. Average value of each trial is given by a filled triangle of the corresponding color. Results are represented as mean ± SD; error bars represent SD. The P values represent a paired two-sample two-tailed *t* test. **(J)** Relative intensity of SPINDLY at the spindle pole in control and ZWILCH-depleted RPE1 cells treated with STLC and GSK923295 overnight. *n* > 60 cells were quantified for each condition. Experiments were performed in triplicate and data from each trial was color-coded. Average value of each trial is given by a filled triangle of the corresponding color. Results are represented as mean ± SD; error bars represent SD. The P values represent a paired two-sample two-tailed *t* test. **(K)** Immunostaining of ZW10 (green), CENP-E (magenta), and CENP-C (blue) in RPE1 cells treated with STLC and GSK923295 overnight in the presence of DMSO or 5 μM lonafarnib. Bar, 5 μm. **(L)** Relative intensity of ZW10 at the spindle pole in RPE1 cells treated with STLC and GSK923295 overnight in the presence of DMSO or 5 μM lonafarnib. *n* > 60 cells were quantified for each condition. Experiments were performed in triplicate and data from each trial was color-coded. Average value of each trial is given by a filled triangle of the corresponding color. Results are represented as mean ± SD; error bars represent SD. The P values represent a paired two-sample two-tailed *t* test.

Having observed that overexpressed 2111-C recruits RZZ-S in interphase cells, we next wished to examine whether endogenous CENP-E interacts with RZZ-S in mitosis. Kinetochores in cells with monopolar spindles (due to treatment with the Eg5 inhibitor S-trityl-L-cysteine [STLC] [Brier et al., 2004]) have small fibrous coronas (likely as a result of ongoing dynein-mediated poleward stripping) with detectable amounts of ZW10 and CENP-E (Fig. 5, A and C; Famulski et al., 2011; Howell et al., 2001; Sacristan et al., 2018). Unexpectedly, additional inhibition of CENP-E with GSK923295 resulted in displacement of CENP-E and ZW10 from kinetochores and accumulation on the spindle pole (Fig. 5, A–C). This polar accumulation was not a result of dynein motor activity (see siZW10 below), but, since motor-inactive CENP-E tends to tightly bind microtubules (Wood et al., 2010), instead may result from transport of the inhibited CENP-E by poleward microtubule flux. Regardless of the exact mechanism, this phenotype provided us with a useful assay to examine potential kinetochore proteins that co-accumulate with CENP-E on spindle poles and to examine the mechanism thereof. Besides CENP-E and ZW10, treatment with GSK923295 caused depletion from kinetochores and accumulation on the spindle pole of the fibrous corona proteins SPINDLY, ZWILCH, MAD1, and p150^Glued^ but not of the outer kinetochore proteins BUBRI and ZWINT-1 (Fig. 5 D and Fig. S3 F). The kinetochore depletion and polar accumulation of ZW10 and ZWILCH, and therefore presumably of the other fibrous corona proteins, depended on CENP-E (Fig. 5, E and F; and Fig. S3, K and L). In contrast, ZW10 RNAi did not impact CENP-E accumulation on the spindle pole (Fig. 5, E and G). Furthermore, ZWILCH depletion prevented SPINDLY accumulation, and vice versa (Fig. 5, H–J), showing that RZZ-S members behaved as one complex. We conclude that in cells with monopolar spindles, transport of inactive CENP-E from kinetochores to spindle poles causes translocation of fibrous corona proteins. Importantly, the ability of inactive CENP-E to translocate RZZ-S was dependent on its C-terminal farnesylation: lonafarnib treatment of RPE1 cells (Fig. 5, K and L; and Fig. S3, G–J) or expression of unfarnesylated mutants of CENP-E in CENP-E–depleted HeLa cells (Fig. S3, K and L) abolished translocation of RZZ-S and MAD1 to spindle poles.

### CENP-E impacts fibrous corona formation after initial RZZ-S kinetochore recruitment

**Figure 6.**
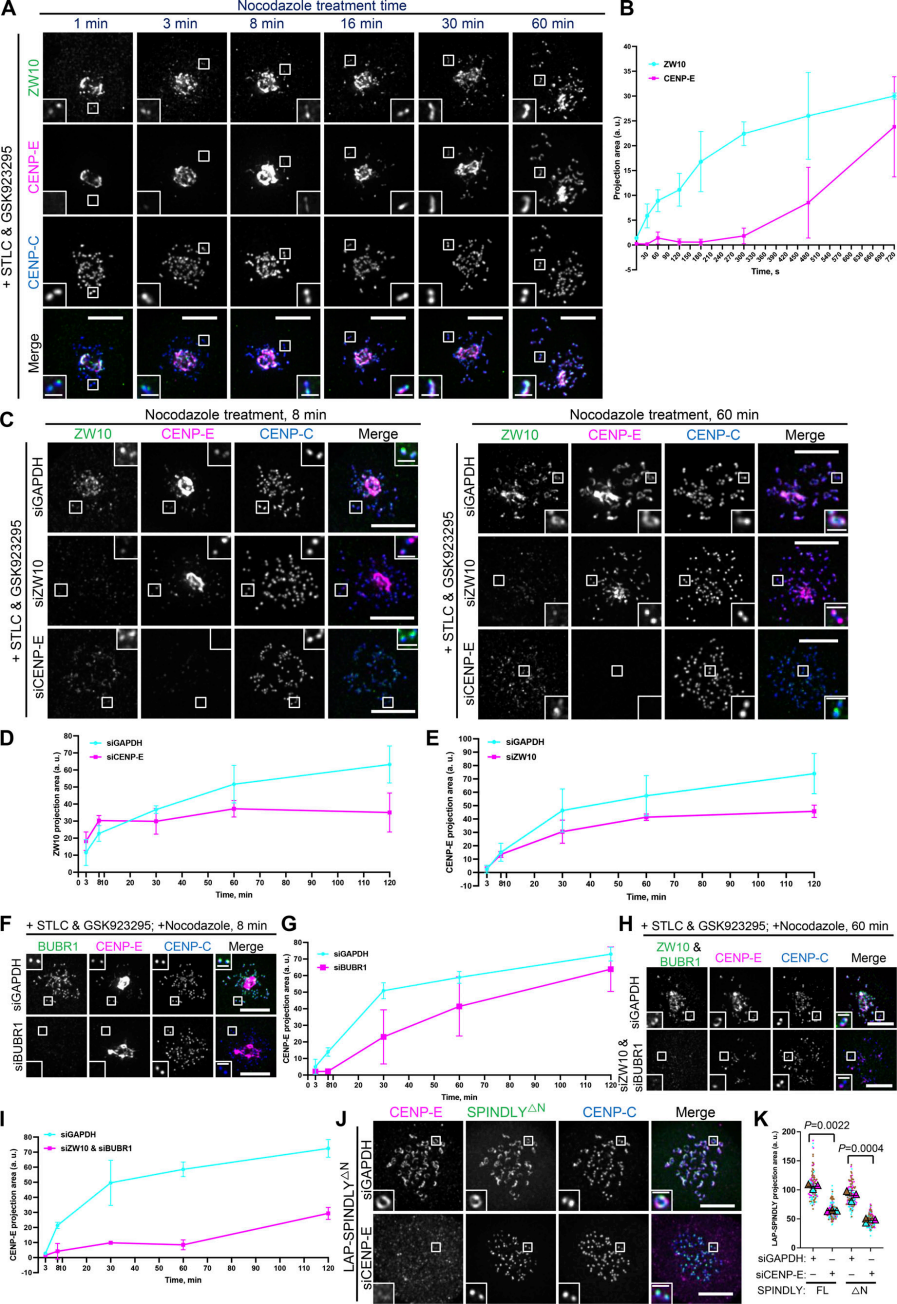
**CENP-E impacts fibrous corona formation after initial RZZ-S kinetochore recruitment. (A)** Immunostaining of ZW10 (green), CENP-E (magenta), and CENP-C (blue) in RPE1 cells treated with nocodazole for indicated time, which had been arrested with STLC and GSK923295 overnight. Bar, 5 μm; inset bar, 1 μm. **(B)** Projection area of ZW10 and CENP-E in RPE1 cells treated with nocodazole for the indicated time, which had been arrested with STLC and GSK923295 overnight. More than 120 pairs of kinetochores were quantified for each condition; two pairs of kinetochores per cell. Experiments were performed in triplicate. Average value of each trial was used to calculate the result. Results are represented as mean ± SD; error bars represent SD. **(C)** Immunostaining of ZW10 (green), CENP-E (magenta), and CENP-C (blue) in control, ZW10-depleted, and CENP-E–depleted RPE1 cells treated with nocodazole for indicated time, which had been arrested with STLC and GSK923295 overnight. Bar, 5 μm; inset bar, 1 μm. **(D)** Projection area of ZW10 in control and CENP-E–depleted RPE1 cells treated with nocodazole for indicated time, which had been arrested with STLC and GSK923295 overnight. More than 120 pairs of kinetochores were quantified for each condition; two pairs of kinetochores per cell. Experiments were performed in triplicate. Average value of each trial was used to calculate the result. Results are represented as mean ± SD; error bars represent SD. **(E)** Projection area of CENP-E in control and ZW10-depleted RPE1 cells treated with nocodazole for indicated time, which had been arrested with STLC and GSK923295 overnight. More than 120 pairs of kinetochores were quantified for each condition; two pairs of kinetochores per cell. Experiments were performed in triplicate. Average value of each trial was used to calculate the result. Results are represented as mean ± SD; error bars represent SD. **(F)** Immunostaining of BUBR1 (green), CENP-E (magenta), and CENP-C (blue) in control and BUBR1-depleted RPE1 cells treated with nocodazole for indicated time, which had been arrested with STLC and GSK923295 overnight. Bar, 5 μm; inset bar, 1 μm. **(G)** Projection area of CENP-E in control and BUBR1-depleted RPE1 cells treated with nocodazole for indicated time, which had been arrested with STLC and GSK923295 overnight. More than 120 pairs of kinetochores were quantified for each condition; two pairs of kinetochores per cell. Experiments were performed in triplicate. Average value of each trial was used to calculate the result. Results are represented as mean ± SD; error bars represent SD. **(H)** Immunostaining of ZW10 (green), BUBR1 (green), CENP-E (magenta), and CENP-C (blue) in control and ZW10 and BUBR1–codepleted RPE1 cells treated with nocodazole for the indicated time, which had been arrested with STLC and GSK923295 overnight. Bar, 5 μm; inset bar, 1 μm. **(I)** Projection area of CENP-E in control and ZW10 and BUBR1–codepleted RPE1 cells treated with nocodazole for indicated time, which had been arrested with STLC and GSK923295 overnight. More than 120 pairs of kinetochores were quantified for each condition; two pairs of kinetochores per cell. Experiments were performed in triplicate. Average value of each trial was used to calculate the result. Results are represented as mean ± SD; error bars represent SD. **(J)** Immunostaining of CENP-E (magenta) and CENP-C (blue) in control and CENP-E–knockdown RPE1 cells that inducibly overexpress LAP-SPINDLY ΔN65 after nocodazole treatment overnight. Bar, 5 μm; inset bar, 1 μm. **(K)** Projection area of LAP-SPINDLY FL/ΔN65 in control and CENP-E–knockdown RPE1 cells that inducibly overexpress LAP-SPINDLY FL/ΔN65 after nocodazole treatment overnight. *n* > 120 pairs of kinetochores were quantified for each condition; two pairs of kinetochores per cell. Experiments were performed in triplicate and data from each trial was color-coded. Average value of each trial is given by a filled triangle of the corresponding color. Results are represented as mean ± SD; error bars represent SD. The P values represent a paired two-sample two-tailed *t* test.

**Figure S4.**
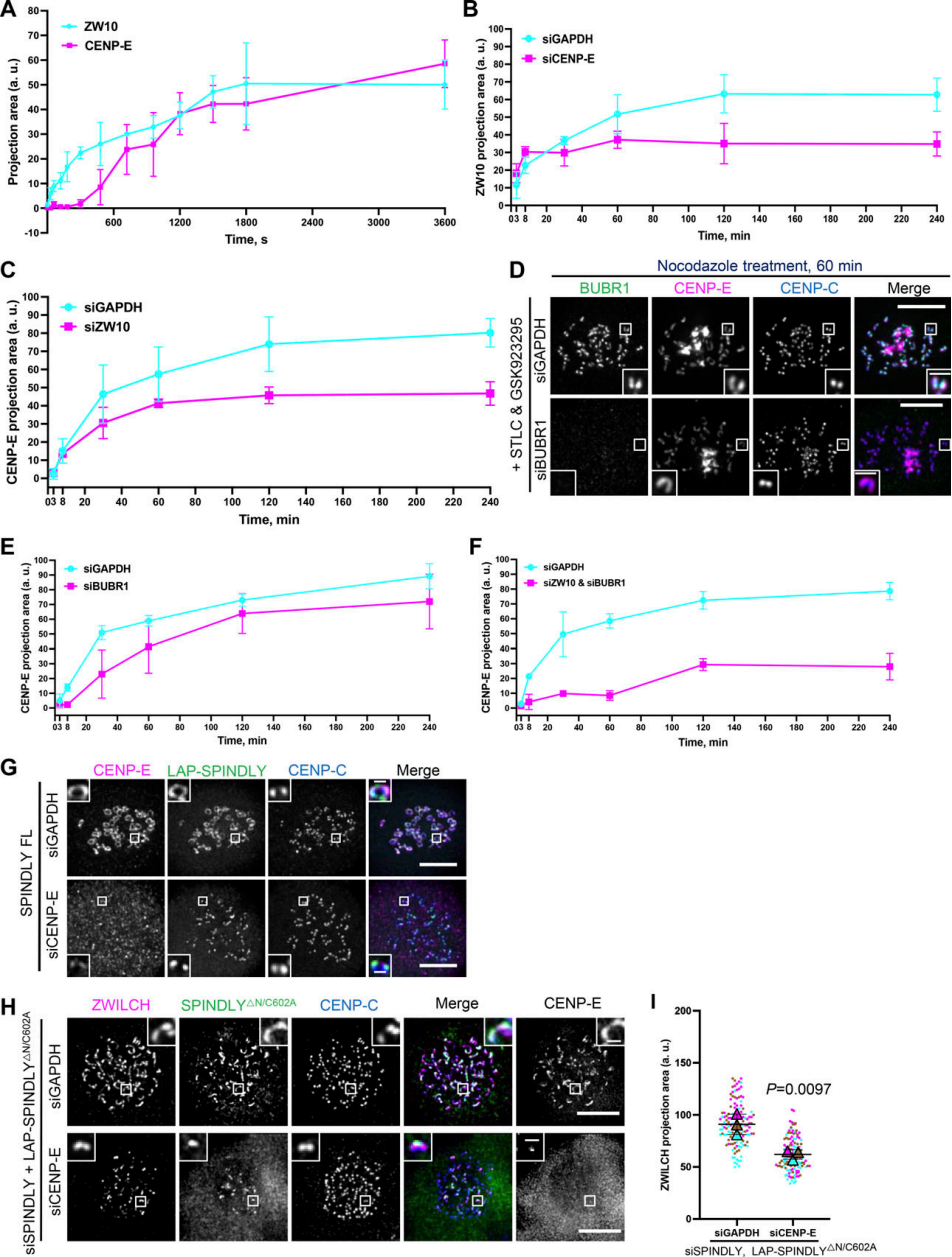
**CENP-E impacts fibrous corona formation after initial RZZ-S kinetochore recruitment. (A)** Projection area of ZW10 and CENP-E in RPE1 cells treated with nocodazole for the indicated time, which had been arrested with STLC and GSK923295 overnight. More than 120 pairs of kinetochores were quantified for each condition; two pairs of kinetochores per cell. Experiments were performed in triplicate. Average value of each trial was used to calculate the result. Results are represented as mean ± SD; error bars represent SD. **(B)** Projection area of ZW10 in control and CENP-E–depleted RPE1 cells treated with nocodazole for indicated time, which had been arrested with STLC and GSK923295 overnight. More than 120 pairs of kinetochores were quantified for each condition; two pairs of kinetochores per cell. Experiments were performed in triplicate. Average value of each trial was used to calculate the result. Results are represented as mean ± SD; error bars represent SD. **(C)** Projection area of CENP-E in control and ZW10-depleted RPE1 cells treated with nocodazole for the indicated time, which had been arrested with STLC and GSK923295 overnight. More than 120 pairs of kinetochores were quantified for each condition; two pairs of kinetochores per cell. Experiments were performed in triplicate. Average value of each trial was used to calculate the result. Results are represented as mean ± SD; error bars represent SD. **(D)** Immunostaining of BUBR1 (green), CENP-E (magenta), and CENP-C (blue) in control and BUBR1-depleted RPE1 cells treated with nocodazole for indicated time, which had been arrested with STLC and GSK923295 overnight. Bar, 5 μm; inset bar, 1 μm. **(E)** Projection area of CENP-E in control and BUBR1-depleted RPE1 cells treated with nocodazole for indicated time, which had been arrested with STLC and GSK923295 overnight. More than 120 pairs of kinetochores were quantified for each condition; two pairs of kinetochores per cell. Experiments were performed in triplicate. Average value of each trial was used to calculate the result. Results are represented as mean ± SD; error bars represent SD. **(F)** Projection area of CENP-E in control and ZW10 and BUBR1–codepleted RPE1 cells treated with nocodazole for indicated time, which had been arrested with STLC and GSK923295 overnight. More than 120 pairs of kinetochores were quantified for each condition; two pairs of kinetochores per cell. Experiments were performed in triplicate. Average value of each trial was used to calculate the result. Results are represented as mean ± SD; error bars represent SD. **(G)** Immunostaining of CENP-E (magenta) and CENP-C (blue) in control and CENP-E–knockdown RPE1 cells that inducibly overexpress LAP-SPINDLY FL after nocodazole treatment overnight. Bar, 5 μm; inset bar, 0.5 μm. **(H)** Immunostaining of ZWILCH (magenta), CENP-E (gray), and CENP-C (blue) in control and CENP-E–depleted HeLa cells that overexpress the LAP-SPINDLY^ΔN^/C602A mutant in the presence of depletion of endogenous SPINDLY after 6 h nocodazole treatment. Bar, 5 μm; inset bar, 0.5 μm. **(I)** Projection area of ZWILCH in control and CENP-E–depleted HeLa cells that overexpress the LAP-SPINDLY^ΔN^/C602A mutant in the presence of depletion of endogenous SPINDLY after 6 h nocodazole treatment. *n* > 120 pairs of kinetochores were quantified for each condition; two pairs of kinetochores per cell. Experiments were performed in triplicate and data from each trial was color-coded. Average value of each trial is given by a filled triangle of the corresponding color. Results are represented as mean ± SD; error bars represent SD. The P value represents a paired two-sample two-tailed *t* test.

Our data thus far showed that farnesylated CENP-E is important for fibrous corona formation and that CENP-E can quite robustly recruit RZZ-S to ectopic locations in a manner dependent on its farnesylation. To understand more about how CENP-E promotes fibrous corona formation, we asked when and how CENP-E localizes to kinetochores, relative to RZZ-S. Again we made use of the monopolar spindle assay, in which fibrous corona proteins are depleted from kinetochores and accumulate at the spindle pole upon chemical inhibition of CENP-E. Subsequent addition of nocodazole to these cells to deplete microtubules caused detectable relocalization of ZW10 back to the kinetochores within ∼1 min, followed by CENP-E after ∼8 min (Fig. 6, A and B; and Fig. S4 A). Importantly, CENP-E depletion did not affect the initial recruitment of ZW10 under these conditions but did impact the ability of ZW10 to expand to the recognizable crescents of the fibrous corona and vice versa (Fig. 6, C–E; and Fig. S4, B and C). CENP-E and ZW10 thus initially localized to kinetochores independently but—perhaps in the context of different pools—required each other for fibrous corona expansion thereafter. CENP-E interacts with BUBR1 (Chan et al., 1998; Ciossani et al., 2018; Legal et al., 2020; Yao et al., 2000), and indeed depletion of BUBR1 abrogated the initial reappearance of CENP-E at kinetochores after nocodazole addition, but not its localization to fibrous coronas at later stages (Fig. 6, F and G; and Fig. S4, D and E). Codepletion of ZW10 and BUBR1 blocked the early recruitment of CENP-E to kinetochores and inhibited subsequent fibrous corona expansion (Fig. 6, H and I; and Fig. S4 F). Together, these data suggest that CENP-E is recruited to kinetochores independently of and after RZZ-S and subsequently promotes RZZ-S–mediated fibrous corona expansion.

SPINDLY has a central role in RZZ-S oligomerization—which in vitro does not require CENP-E (Raisch et al., 2022; Sacristan et al., 2018)—that involves a conformational transition to relieve auto-inhibition (Sacristan et al., 2018). A conformationally “open” SPINDLY (SPINDLY^ΔN^) bypasses the need for regulatory mechanisms of fibrous corona formation and does not bind dynein/dynactin, resulting in persistent fibrous coronas (Raisch et al., 2022; Sacristan et al., 2018). If CENP-E functions upstream of SPINDLY conformational “activation,” expression of SPINDLY^ΔN^ should restore fibrous coronas in CENP-E–depleted cells. As shown in Fig. 6, J and K, and Fig. S4, G–I, fibrous corona formation in SPINDLY^ΔN^-expressing cells still relied on CENP-E, and this was true also when SPINDLY could not be farnesylated (SPINDLY^ΔN/C602A^; Sacristan et al., 2018). The contribution of CENP-E to fibrous corona expansion is therefore unrelated to the mechanisms that control SPINDLY conformation and is independent of SPINDLY farnesylation. Given that RZZ-S can oligomerize without CENP-E (Raisch et al., 2022; Sacristan et al., 2018), these data suggest CENP-E impacts the fibrous corona in other ways.

### Comparative genomics of fibrous corona formation mechanisms in eukaryotes

**Figure S5.**
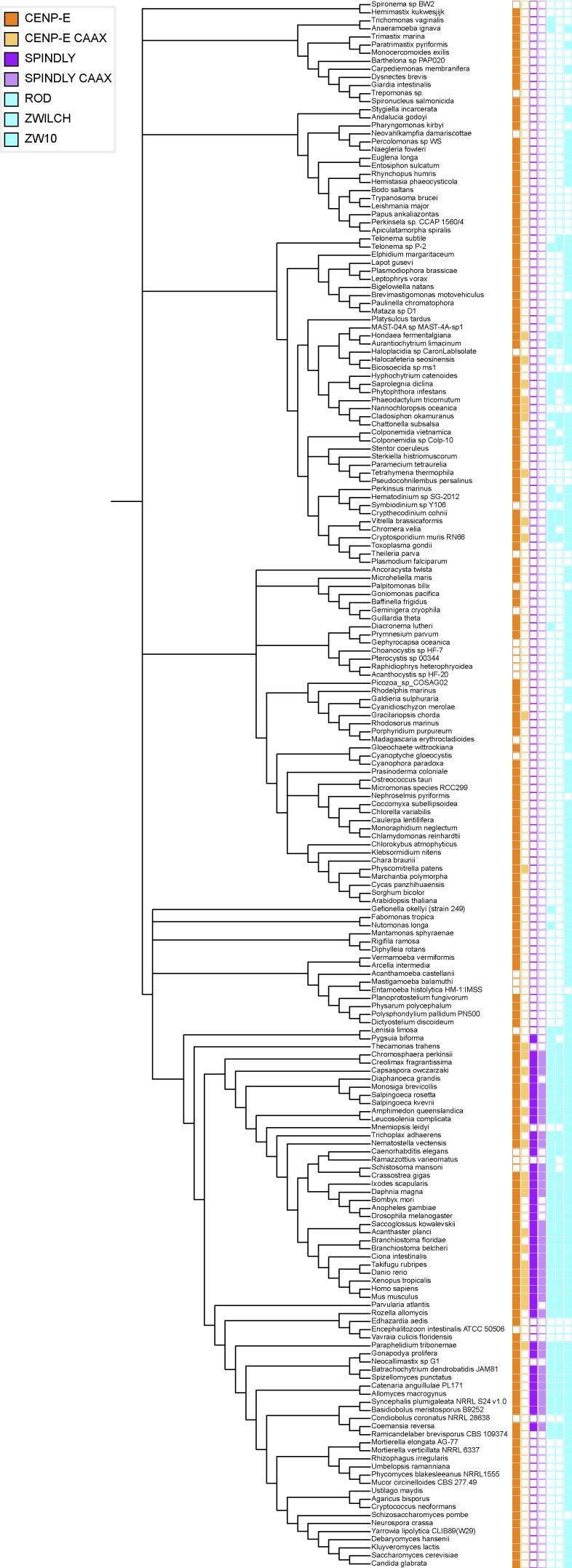
**Comparative genomics of fibrous corona formation mechanisms in eukaryotes.** Phylogenetic profiling of CENP-E, the CAAX box of CENP-E, SPINDLY, the CAAX box of SPINDLY, ROD, ZWILCH, and ZW10 across the entire database representing eukaryotic diversity (194 species). Presences are represented with colored squares, with absences indicated by white squares.

**Figure 7.**
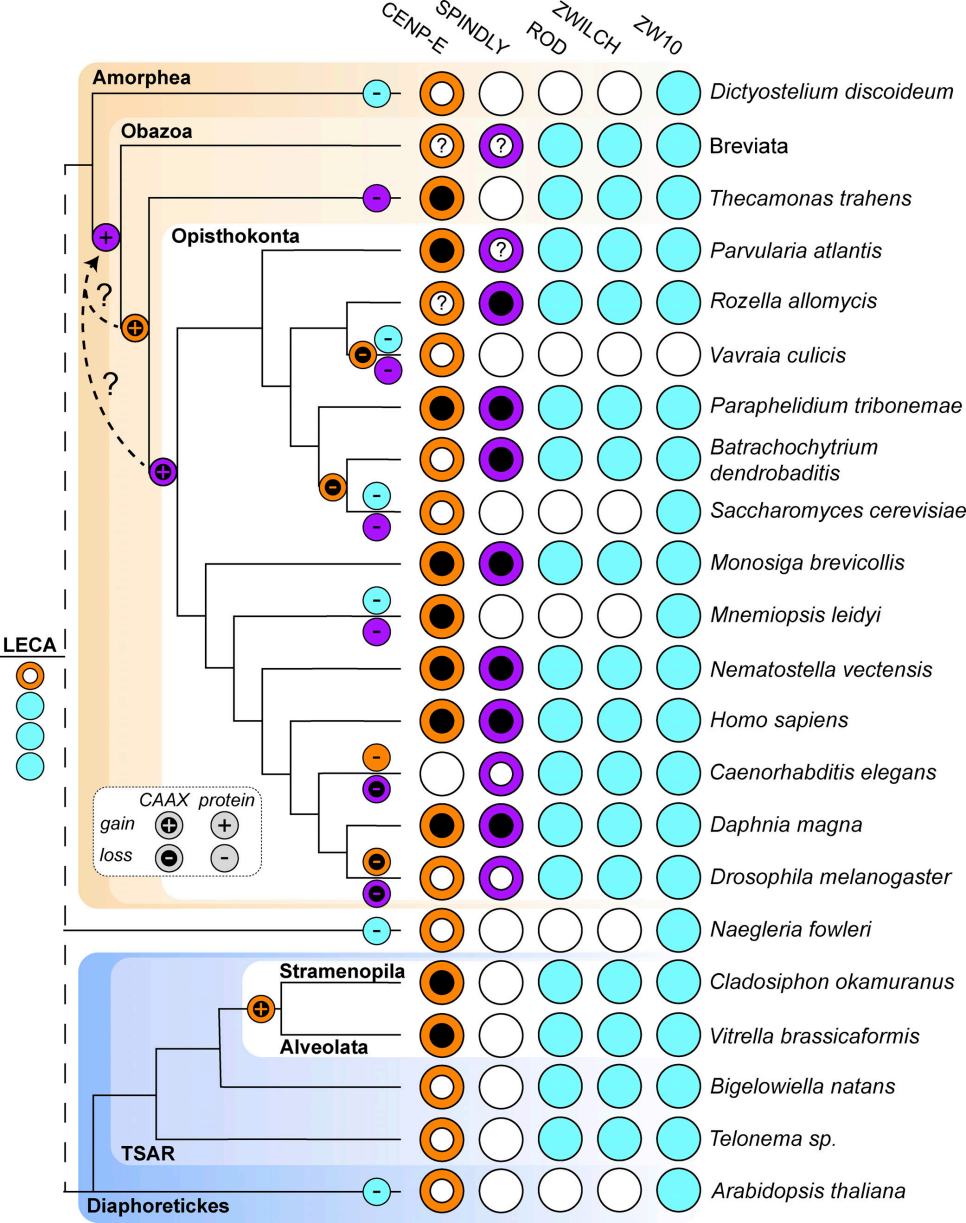
**Comparative genomics of fibrous corona formation mechanisms in eukaryotes.** Phylogenetic profiling of CENP-E, the CAAX box of CENP-E, SPINDLY, the CAAX box of SPINDLY, ROD, ZWILCH, and ZW10 across eukaryotic diversity represented by selected species. Evolutionary reconstructions of the loss and gain of proteins and CAAX boxes are projected onto the phylogenetic tree on the left-hand side of the figure. Presences are represented by colored circles and absences are represented by white circles. Due to the poor quality of the sequence data in the Breviata, the presence/absence data is the combination of inferences of two member species (*Pygsuia biforma* and *Lenisia limosa*). For inferring the presence of a CAAX box in either CENP-E or SPINDLY, having a complete protein sequence is essential. In cases where features of the predicted protein sequence strongly suggest that only a fragment is available, it is not possible to infer the presence of a CAAX box, and such cases are indicated with a question mark (?) in a white internal CAAX circle. These fragments affect the inference of the exact timing of the CENP-E and SPINLY CAAX boxes, which emerged at the latest in the last common ancestor of Opisthokonta but may have already been present in the ancestor of the Obazoa, as indicated with the dotted line with a question mark. LECA: Last Eukaryotic Common Ancestor.

To examine whether the mechanisms for fibrous corona formation—including the contribution of farnesylated CENP-E that we report here—might be conserved in eukaryotes, we built on our previous approach of comparative genomics of diverse eukaryotic species (van Hooff et al., 2017). While ZW10 orthologs are omnipresent in eukaryotes, likely due to their primary role in vesicular trafficking (Sun et al., 2007), RZS (ROD/ZWILCH/SPINDLY) is an evolutionary coherent module in eukaryotes: there is a high degree of co-occurrence (presence or absence) of orthologs of its members in eukaryotic species, with co-presence of all RZZ-S complex members being restricted to Opisthokonta (fungi and animals; van Hooff et al., 2017). We strengthened these previous observations in a larger, more diverse set of species genomes, utilizing refined homology detection methods (Fig. S5). Given that RZZ-S form a biochemical unit in several animal model organisms, its coherent presence/absence profiles in eukaryotic species could signal the existence of a similar functional unit, and perhaps even of fibrous coronas, in the various species whose genomes encode RZZ-S orthologs. In contrast to RZS, CENP-E orthologs can be found in many species from all major eukaryotic lineages, similar to ZW10 (Fig. 7; van Hooff et al., 2017). However, CENP-E orthologous genes encoding a C-terminal CAAX box (defined by cysteine at the −4 position from the C-terminus) appear only in two distantly related groups: the Opisthokonta/Apusozoa, which unites fungi, animals, and several unicellular relatives, and the Stramenopila/Alveolata (Fig. 7 and Fig. S5). Due to the large phylogenetic distance between these lineages, we infer that the acquisitions of CAAX box in CENP-E ancestors were likely independent. Importantly, SPINDLY is inferred to emerge at the base of Obazoa (Opisthokonta/Apusozoa/Breviata), and in this lineage, CENP-E’s CAAX box has a high degree of co-occurrence with RZS orthologs (Fig. 7 and Fig. S5). Emergence of the CAAX box in CENP-E near the base of the Obazoa therefore coincides with the emergence of SPINDLY and with the presence of the full RZZ-S unit.

## Discussion

**Figure 8.**
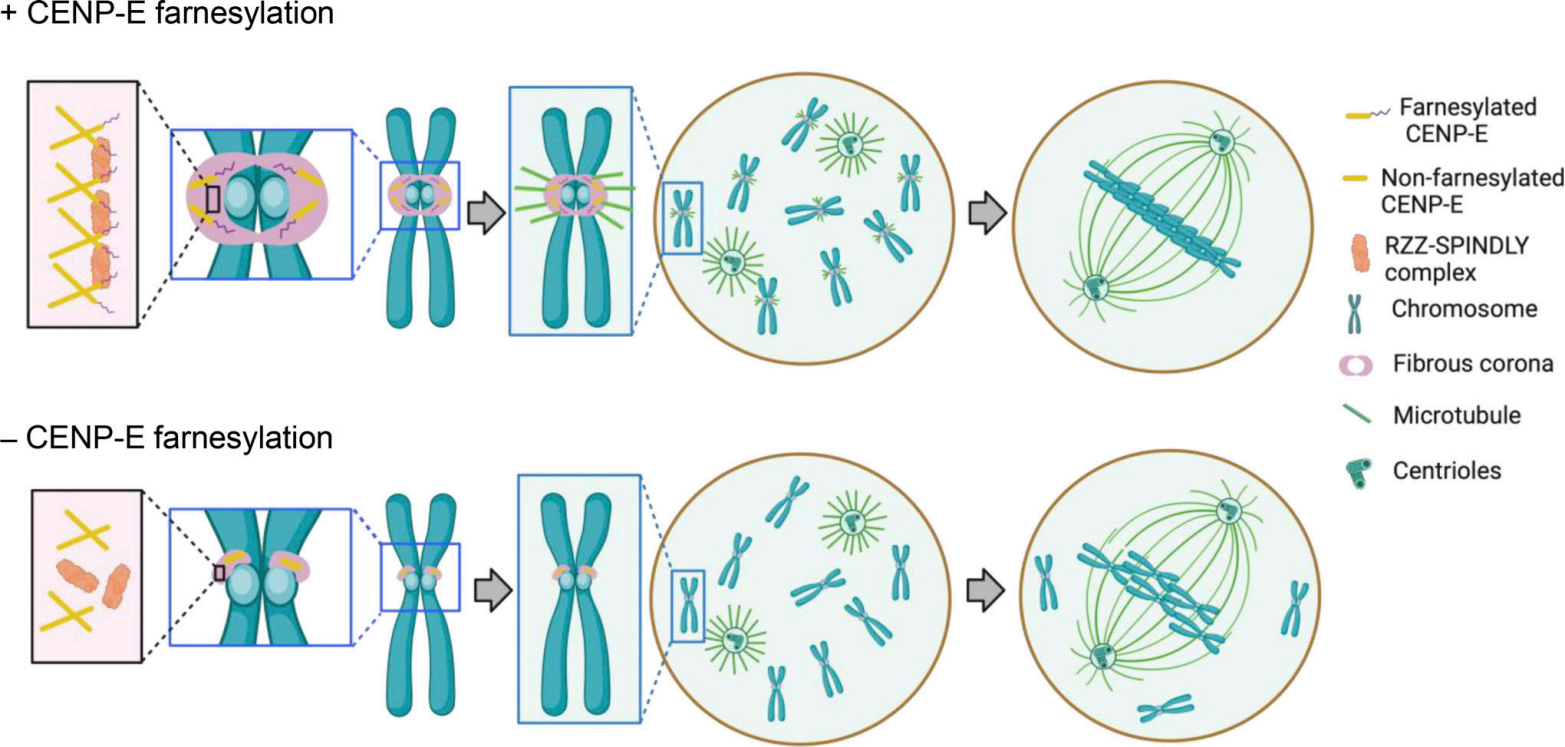
**Model.** Cartoon model showing the association between CENP-E and RZZ-SPINDLY, fibrous corona status, and related functions in terms of kinetochore-based microtubule nucleation and chromosome alignment as consequences of presence/absence of farnesylation of CENP-E.

The fibrous corona is a meshwork that temporarily covers the outer kinetochores during early mitosis and meiosis and assists in spindle assembly (Kops and Gassmann, 2020). Several recent studies have shown key mechanisms for how it expands and compacts. Central to these mechanisms is the RZZ-S complex, which can oligomerize into a filamentous meshwork in vitro (Pereira et al., 2018; Raisch et al., 2022; Sacristan et al., 2018). In cells, this oligomerization requires MPS1 activity and a conformational transition of SPINDLY, involving release of autoinhibition imposed by N-terminal sequences (Raisch et al., 2022; Sacristan et al., 2018). We now show that fibrous corona formation in cells additionally requires the mitotic kinesin CENP-E. This does not involve its motor activity since both chemically inhibited CENP-E and truncated versions of CENP-E lacking the motor domain can substitute for endogenous CENP-E in supporting fibrous corona formation. Instead, its C-terminal ∼590 amino acids and farnesylation of cysteine 2698 suffice for this newly identified function. As such, CENP-E’s contributions to error-free chromosome segregations broaden: through its involvement in fibrous corona formation, CENP-E contributes to kinetochore-derived microtubule nucleation, spindle assembly, and chromosome congression (Fig. 8).

Given that RZZ-S oligomerization is the driving mechanism for fibrous corona formation, it is likely that CENP-E impinges on this process. Our proteomics analysis of CENP-E–interacting proteins and our observations that CENP-E recruits RZZ-S to ectopic sites in interphasic and mitotic cells suggests that CENP-E interacts—directly or indirectly—with RZZ-S, possibly via SPINDLY. The CENP-E/RZZ-S interaction is most likely mediated by the KT-binding domain of CENP-E: it is included in the 2111-C fragment and is essential for binding of CENP-E to kinetochores and fibrous coronas (Chan et al., 1998). The interaction additionally requires farnesylation of CENP-E, and this is also critical for fibrous corona formation. It is noteworthy that SPINDLY^ΔN^ partially rescues fibrous corona expansion in the absence of global farnesylation (Sacristan et al., 2018), but not in the absence of CENP-E (Fig. 6 J). This suggests that CENP-E’s role in fibrous corona expansion is not solely mediated by CENP-E farnesylation and may include additional interactions with RZZ-S.

Our findings uncover a new role for the mitotic kinesin CENP-E and thereby expand the factors known to be required for fibrous corona formation and kinetochore function in spindle assembly. How does CENP-E contribute to fibrous corona formation? It could affect the regulation of RZZ-S oligomerization by MPS1, involving release of the autoinhibition of SPINDLY (Raisch et al., 2022; Sacristan et al., 2018). However, if this were true, persistently uninhibited SPINDLY (SPINDLY^ΔN^) should be able to bypass this step in the absence of CENP-E. This is not so, showing CENP-E contributes to a parallel or downstream event. An alternative hypothesis is that CENP-E is an integral component of the RZZ-S meshwork. However, RZZ-S can oligomerize in vitro without CENP-E (Raisch et al., 2022; Sacristan et al., 2018). In kinetochores, however, the molecular environment is different, and CENP-E might be required to promote the reaction, for example, as a catalyst, although there is currently no obvious molecular rationale for this. Based on our observation that CENP-E follows RZZ to kinetochores, perhaps a more likely model is one in which CENP-E stabilizes the RZZ-S meshwork during its expansion (Fig. 8). SPINDLY interacts with RZZ via its farnesyl moiety that docks into a hydrophobic pocket in the ROD β-propeller (Mosalaganti et al., 2017; Raisch et al., 2022). A similar mode of interaction may apply to farnesylated CENP-E. This could be through the known farnesyl binding pocket on ROD or a different region in the RZZ-S complex, for example, SPINDLY, which was the RZZ-S member most proximal to farnesylated 2111-C in our proximity biotinylation experiments. Extensive biochemical reconstitution experiments with RZZ-S and CENP-E under different conditions are needed to reveal the exact molecular mechanism.

How conserved is the CENP-E contribution we identified in the present study? The fibrous corona structure as defined by electron microscopy imaging of unattached kinetochores has been observed in cells of various vertebrates (Bielek, 1978; Jokelainen, 1967; Rieder, 1982; Roos, 1973), but its existence has not been examined extensively in other lineages (e.g., invertebrate animals, plants, or fungi). Our deep homology searches for homologs of RZZ-S members revealed that the set of genes encoding the full complex emerges at the base of the Obazoa, with the emergence of ancestral SPINDLY. The core ingredients for fibrous corona formation were thus present in the ancestor of Obazoa. Although CENP-E orthologs are widespread in eukaryotes, the appearance of a farnesyl-acceptor cysteine residue at position −4 from the carboxy terminus in the Obazoa lineage shows a striking correlation with the emergence of SPINDLY (and its CAAX motif) and thus the full RZZ-S complex (of note: farnesyl transferase activity is ubiquitous in eukaryotes [see KOG0365 at http://eggnog6.embl.de], including in all species here identified as having CAAX motifs in CENP-E or Spindly). Our current analysis pinpoints emergence of SPINDLY one branch earlier than the cysteine in CENP-E, but we note that the CENP-E ortholog sequences in Breviata are incomplete. Better sampling of genomes from Breviata species might therefore reveal FL CENP-E ORF sequences with a cysteine at −4 from the carboxy terminus, which would indicate near-simultaneous emergence of SPINDLY and the potential for CENP-E farnesylation. Beyond SPINDLY, the RZZ-S complex and the potential for CENP-E farnesylation show strongly cohesive presence/absence in Obazoa, with notable coherent losses close in the species tree. Interestingly, kinetochores in colchicine-treated *Drosophila* S2 cells do not appear to form clear fibrous coronas (Maiato et al., 2006) and their CENP-E and SPINDLY orthologs lack CAAX boxes. Our analyses revealed emergence of a cysteine at −4 in CENP-E also in the Stramenopiles/Alveolate lineage of eukaryotes. Given the simple trajectory to acquiring a single amino acid change and given the absence of the cysteine in all other lineages except Obazoa, it was likely acquired independently in the Stramenopiles/Alveolate lineage. Species in this lineage also have RZZ orthologs, raising the question of whether they may have the capacity to form a structure analogous to a fibrous corona, despite the apparent absence of a SPINDLY ortholog. Future cell biological and biochemical studies on these and other species will be important to understand conservation and diversity in fibrous corona form and function among eukaryotes.

## Materials and methods

### Cell culture

RPE1 FlpIn cells, HEK293T, and HeLa FlpIn cells were cultured in Dulbecco’s Modified Eagle Medium/Nutrient Mixture F-12 (DMEM/F-12 GlutaMAX; 10565018; Gibco) supplemented with 9% tetracycline-free fetal bovine serum and 100 μg/ml penicillin-streptomycin (P0781; Sigma-Aldrich).

### Plasmids and cloning

LAP-SPINDLY FL, LAP-SPINDLY^ΔN^, and LAP-SPINDLY^ΔN/C602A^ in pCDNA5 vector were previously cloned by PCR-based strategy; SPINDLY^ΔN^: SPINDLY 65-605; SPINDLY^ΔN/C602A^: SPINDLY 65-605 with C602A (Sacristan et al., 2018). LAP–CENP-E full length and mutants in pCDNA5 vector were cloned by PCR-based strategy. Mutant C-10A1: CENP-E 2111-C with amino acids from 2,600 to 2,609 substituted for 10 alanine residues; mutant C-10A2: CENP-E 2111-C with amino acids from 2,610 to 2,619 substituted for 10 alanine residues; mutant C-10A3: CENP-E 2111-C with amino acids from 2,620 to 2,629 substituted for 10 alanine residues; mutant C-10A4: CENP-E 2111-C with amino acids from 2,630 to 2,639 substituted for 10 alanine residues; mutant C-10A5: CENP-E 2111-C with amino acids from 2,640 to 2,649 substituted for 10 alanine residues; mutant C-10A6: CENP-E 2111-C with amino acids from 2,650 to 2,659 substituted for 10 alanine residues; mutant C-10A7: CENP-E 2111-C with amino acids from 2,660 to 2,669 substituted for 10 alanine residues; mutant C-10A8: CENP-E 2111-C with amino acids from 2,670 to 2,679 substituted for 10 alanine residues; mutant C-10A9: CENP-E 2111-C with amino acids from 2,680 to 2,689 substituted for 10 alanine residues; and mutant 2,111–2,689: CENP-E 2,111–2,689.

LAP-tagged CENP-E 1–426^2111-C mutants were generated by fusing the N-terminal CENP-E 1–426 (comprising the motor domain) to the C-terminal CENP-E 2111-C (comprising KT-binding domain and MT-binding domain). LAP-tagged CENP-E 2,111–2,697^CAAX^KRAS4b^ was generated by fusing the CENP-E 2,111–2,697 to the CAAX box of KRAS4b.

LAP-ROD in pCDNA5 vector was cloned based on the ROD cDNA, which was a kind gift from Reto Gassmann (Instituto de Biologia Molecular e Celular, Porto, Portugal), TagRFP-CENP-E 2111-C and H2B-mScarlet were cloned into the pLVX-IRES-Puro vector (Clontech) by PCR-based Gibson assembly method.

### Primers

CENP-E-1aa-XhoI-GA-F: 5′-CGG​AGG​TGG​ATC​CAC​TAG​TCT​CGA​GAT​GGC​GGA​GGA​AGG​AGC​CGT-3′
CENP-E-2,701aa-NotI-GA-R: 5′-GCG​GGT​TTA​AAC​GGG​CCC​GCG​GCC​GCC​TAC​TGA​GTT​TTG​CAC​TCA​GGC​ACA​TC-3′
CENP-E-1,301-BamHI-F: 5′-GCA​CAT​GGA​CAG​CGG​AGG​TGG​ATC​CAC​TCA​GGA​AAC​AAT​GAA​TGA​ACT​GGA​GT-3′
CENP-E-2,111-BamHI-F: 5′-GCA​CAT​GGA​CAG​CGG​AGG​TGG​ATC​CGA​TGA​TCA​TTA​TGA​GTG​CTT​GAA​TAG​ATT​GTC​TCT-3′
CENP-E 2,599-NotI-R: 5′-GCG​GGT​TTA​AAC​GGG​CCC​GCG​GCC​GCC​TAT​TGT​TTG​TGA​GCC​TCT​CTT​TTA​AGG​GTT-3′
CENP-E 2,643-NotI-R: 5′-GCG​GGT​TTA​AAC​GGG​CCC​GCG​GCC​GCC​TAA​CAA​GAT​TTT​GGT​GAT​TCC​TTT​GGC​ACA-3′
CENP-E-2,600-mutation10AA-GA-R: 5′-TGC​AGC​CGC​TGC​AGC​TGC​CGC​TGC​GGC​AGC​TTG​TTT​GTG​AGC​CTC​TCT​TTT​AAG​GGT​T-3′
CENP-E-2,609-mutation10AA-GA-F: 5′-GCG​GCA​GCT​GCA​GCG​GCT​GCA​AAA​GTG​ACT​GGA​ACA​GCT​TCT​AAA​AAG​AAA​C-3′
CENP-E-2,610-mutation10AA-GA-R: 5′-TGC​AGC​CGC​TGC​AGC​TGC​CGC​TGC​GGC​AGC​AGG​AGA​CTT​TGG​AGA​ATT​CTC​ACA​AG-3′
CENP-E-2,619-mutation10AA-GA-F: 5′-GCG​GCA​GCT​GCA​GCG​GCT​GCA​CAA​ATT​ACA​CCC​TCT​CAA​TGC​AAG​GAA​C-3′
CENP-E-2,620-mutation10AA-GA-R: 5′-TGC​AGC​CGC​TGC​AGC​TGC​CGC​TGC​GGC​AGC​TTT​CTT​TTT​AGA​AGC​TGT​TCC​AGT​CAC-3′
CENP-E-2,629-mutation10AA-GA-F: 5′-GCG​GCA​GCT​GCA​GCG​GCT​GCA​AAT​TTA​CAA​GAT​CCT​GTG​CCA​AAG​GAA​TC-3′
CENP-E-2,630-mutation10AA-GA-R: 5′-TGC​AGC​CGC​TGC​AGC​TGC​CGC​TGC​GGC​AGC​CCG​TTC​CTT​GCA​TTG​AGA​GGG​TG-3′
CENP-E-2,639-mutation10AA-GA-F: 5′-GCG​GCA​GCT​GCA​GCG​GCT​GCA​CCA​AAA​TCT​TGT​TTT​TTT​GAT​AGC​CGA​TC-3′
CENP-E-2,640-mutation10AA-GA-R: 5′-TGC​AGC​CGC​TGC​AGC​TGC​CGC​TGC​GGC​AGC​TGA​TTC​CTT​TGG​CAC​AGG​ATC​TTG-3′
CENP-E-2,649-mutation10AA-GA-F: 5′-GCG​GCA​GCT​GCA​GCG​GCT​GCA​AAG​TCT​TTA​CCA​TCA​CCT​CAT​CCA​G-3′
CENP-E-2,650-mutation10AA-GA-R: 5′-TGC​AGC​CGC​TGC​AGC​TGC​CGC​TGC​GGC​AGC​TGA​TCG​GCT​ATC​AAA​AAA​ACA​AGA​TTT​TG-3′
CENP-E-2,659-mutation10AA-GA-F: 5′-GCG​GCA​GCT​GCA​GCG​GCT​GCA​TAT​TTT​GAT​AAC​TCA​AGT​TTA​GGC​CTT​TGT​C-3′
CENP-E-2,660-mutation10AA-GA-R: 5′-TGC​AGC​CGC​TGC​AGC​TGC​CGC​TGC​GGC​AGC​GCG​AAC​TGG​ATG​AGG​TGA​TGG​TAA-3′
CENP-E-2,669-mutation10AA-GA-F: 5′-GCG​GCA​GCT​GCA​GCG​GCT​GCA​CCA​GAG​GTG​CAA​AAT​GCA​GGA​G-3′
CENP-E-2,670-mutation10AA-GA-R: 5′-TGC​AGC​CGC​TGC​AGC​TGC​CGC​TGC​GGC​AGC​ACA​AAG​GCC​TAA​ACT​TGA​GTT​ATC​AAA​AT-3′
CENP-E-2,679-mutation10AA-GA-F: 5′-GCG​GCA​GCT​GCA​GCG​GCT​GCA​GTG​GAT​TCT​CAG​CCA​GGT​CCT​TG-3′
CENP-E-2,680-mutation10AA-GA-R: 5′-TGC​AGC​CGC​TGC​AGC​TGC​CGC​TGC​GGC​AGC​ACT​CTC​TGC​TCC​TGC​ATT​TTG​C-3′
CENP-E-2,689-mutation10AA-GA-F: 5′-GCG​GCA​GCT​GCA​GCG​GCT​GCA​TCC​TCA​GGC​AAG​GAT​GTG​CCT​G-3′
CENP-E-2,689-NotI-R: 5′-GCG​GGT​TTA​AAC​GGG​CCC​GCG​GCC​GCC​TAG​GCG​TGC​CAA​GGA​CCT​GGC​TGA​G-3′
CENP-E-2,701-NotI-S2690A-R2: 5′-GCG​GGT​TTA​AAC​GGG​CCC​GCG​GCC​GCC​TAC​TGA​GTT​TTG​CAC​TCA​GGC​ACA​TCC​TTG​CCT​GAA​GCG​GCG​TGC​CAA​GGA​CCT​G-3′
CENP-E-2,701-NotI-S2691A-R2: 5′-GCG​GGT​TTA​AAC​GGG​CCC​GCG​GCC​GCC​TAC​TGA​GTT​TTG​CAC​TCA​GGC​ACA​TCC​TTG​CCA​GCG​GAG​GCG​TGC​CAA​GGA​C-3′
CENP-E-2,701-NotI-K2693A-R2: 5′-GCG​GGT​TTA​AAC​GGG​CCC​GCG​GCC​GCC​TAC​TGA​GTT​TTG​CAC​TCA​GGC​ACA​TCA​GCG​CCT​GAG​GAG​GCG​TGC​CAA​GGA​C-3′
CENP-E-2,701-NotI-D2694A-R2: 5′-GCG​GGT​TTA​AAC​GGG​CCC​GCG​GCC​GCC​TAC​TGA​GTT​TTG​CAC​TCA​GGC​ACA​GCC​TTG​CCT​GAG​GAG​GCG​TGC​CAA​GGA​C-3′
CENP-E-2,701-NotI-V2695A-R: 5′-GCG​GGT​TTA​AAC​GGG​CCC​GCG​GCC​GCC​TAC​TGA​GTT​TTG​CAC​TCA​GGA​GCA​TCC​TTG​CCT​GAG​GAG​GCG​TG-3′
CENP-E-2,701-NotI-P2696A-R: 5′-GCG​GGT​TTA​AAC​GGG​CCC​GCG​GCC​GCC​TAC​TGA​GTT​TTG​CAC​TCA​GCC​ACA​TCC​TTG​CCT​GAG​GAG​GC-3′
CENP-E-2,701-NotI-E2697A-R: 5′-GCG​GGT​TTA​AAC​GGG​CCC​GCG​GCC​GCC​TAC​TGA​GTT​TTG​CAA​GCA​GGC​ACA​TCC​TTG​CCT​GAG​GAG-3′
CENP-E-C-C2698A-R2: 5′-GCG​GGT​TTA​AAC​GGG​CCC​GCG​GCC​GCC​TAC​TGA​GTT​TTA​GCC​TCA​GGC​ACA​TCC​TTG​CCT​GAG​GAG​GC-3′
CENP-E-2,701-NotI-K2699A-R: 5′-GCG​GGT​TTA​AAC​GGG​CCC​GCG​GCC​GCC​TAC​TGA​GTA​GCG​CAC​TCA​GGC​ACA​TCC​TTG​CC-3′
CENP-E-2,701-NotI-T2700A-R: 5′-GCG​GGT​TTA​AAC​GGG​CCC​GCG​GCC​GCC​TAC​TGA​GCT​TTG​CAC​TCA​GGC​ACA​TCC​TTG​C-3′
CENP-E-2,701-NotI-Q2701A-R: 5′-GCG​GGT​TTA​AAC​GGG​CCC​GCG​GCC​GCC​TAA​GCA​GTT​TTG​CAC​TCA​GGC​ACA​TCC​T-3′
CENP-E-2,701-NotI-C2698S-R: 5′-GCG​GGT​TTA​AAC​GGG​CCC​GCG​GCC​GCC​TAC​TGA​GTT​TTT​GAC​TCA​GGC​ACA​TCC​TTG​CCT​GAG​GAG​GCG​TG-3′
CENP-E^KRAS_caax_R: 5′-GCG​GGT​TTA​AAC​GGG​CCC​GCG​GCC​GCC​TAC​ATT​ATA​ATG​CAC​TCA​GGC​ACA​TCC​TTG​CCT​GAG​GAG​GCG​TG-3′
TagRFP-kozak-EcoR1-F: 5′-CTA​CTA​GAG​GAT​CTA​TTT​CCG​GTG​AAT​TCG​CCA​CCA​TGG​TGT​CTA​AGG​GCG​AAG​AGC​TGA​TTA​AG-3′
TagRFP-C-toCENP-E2111-R: 5′-TCT​ATT​CAA​GCA​CTC​ATA​ATG​ATC​ATC​GGA​TCC​ACC​TCC​GCT​ATT​AAG​TTT​GTG​CCC​CAG​TTT​GCT-3′
ROD-1aa-XhoI-GA-F: 5′-CGG​AGG​TGG​ATC​CAC​TAG​TCT​CGA​GAT​GTG​GAA​TGA​TAT​TGA​GCT​GCT​AAC​AAA​TG-3′
ROD-1,100aa-overlap-GA-R: 5′-AAT​AGC​AAT​TTC​CCA​GTG​TCA​GCA​TT-3′
ROD-1,100aa-overlap-GA-F: 5′-AAT​GCT​GAC​ACT​GGG​AAA​TTG​CTA​TT-3′
ROD-2,209aa-NotI-GA-R: 5′-GCG​GGT​TTA​AAC​GGG​CCC​GCG​GCC​GCT​TAC​GAT​AAT​CCA​CTA​AGA​AAC​ATC​TTC​AGA​AT-3′
TurboID-N-BamHI-F: 5′-GCA​CAT​GGA​CAG​CGG​AGG​TGG​ATC​CAA​AGA​CAA​TAC​TGT​GCC​TCT​GAA​GCT-3′
TurboID-C-BamHI-R: 5′-TCA​AGC​ACT​CAT​AAT​GAT​CAT​CGG​ATC​CGC​TGA​ATT​CCT​TTT​CGG​CAG​ACC​GCA​GAC​T-3′
CENP-E-1aa-BamHI-cut-GA-F: 5′-GCA​CAT​GGA​CAG​CGG​AGG​TGG​ATC​CAT​GGC​GGA​GGA​AGG​AGC​CGT​G-3′
CENP-E-426aa-BamHI-cut-GA-R: 5′-ATT​CAA​GCA​CTC​ATA​ATG​ATC​ATC​GGA​TCC​GCC​AAG​GCA​CCA​AGT​AAC​TCT​TC-3′

### Virus production

Lentiviruses were produced by cotransfection of HEK 293T cells with the lentiviral vector containing TagRFP-CENP-E 2111-C or H2B-mScarlet and separate plasmids that express Tat, vesicular stomatitis virus G glycoprotein, Rev, and Gag-Pol, with the transfection reagent of Fugene HD (E2311; Promega). 72 h after transfection, cell culture supernatant was harvested and filtered.

### Stable cell lines and generation

RPE1 FlpIn cell line stably expressing EB3-TagRFPT-ires-GFP-ZWILCH was made previously by transduction of viruses that were produced by cotransfection of HEK 293T cells with the lentiviral vector containing gene of EB3-TagRFPT-ires-GFP-Zwilch, which was cloned into the pLVX-IRES-Puro vector (Wu et al., 2023).

RPE1 FlpIn cell line inducible expressing LAP-SPINDLY FL and LAP- SPINDLY^ΔN^ were made previously by cotransfection of pCDNA5-constructs with gene of interest and construct of pOG44 recombinase in a 1:2 ratio with electroporation machine (Amaxa Nucleofector II) using protocol U-017. 3 d after transfection, RPE1 cells were selected with 100 μM hygromycin (10843555001; Roche) for about 3 wk (Wu et al., 2023).

RPE1 FlpIn cell lines were cotransfected with pCDNA5-constructs with gene of interest and construct of pOG44 recombinase in a 1:2 ratio with an electroporation machine (Amaxa Nucleofector II) using protocol U-017. 3 d after transfection, RPE1 cells were selected with 100 μM hygromycin (10843555001; Roche) for about 3 wk.

HeLa FlpIn were cotransfected with pCDNA5 constructs with the gene of interest and construct of pOG44 recombinase in a 1:9 ratio with Fugene HD (E2311; Promega) under the manufacturer’s instructions. 1 d after transfection, HeLa cells were selected with 200 μM hygromycin (10843555001; Roche) for about 3 wk.

To generate HeLa FlpIn cells stably expressing TagRFP-CENP-E 2111-C and inducible expressing LAP-ROD, HeLa FlpIn cells inducible expressing LAP-ROD were infected with lentiviruses and selected with 1 μg/ml puromycin for 1 wk.

To generate HeLa FlpIn cell stably expressing H2B-mScarlet, HeLa FlpIn were infected with lentiviruses and selected with 1 μg/ml puromycin for 1 wk.

To generate HeLa FlpIn cells inducible expressing CENP-E FL WT or CENP-E FL C2698A and stably expressing H2B-mScarlet, HeLa FlpIn cells inducible expressing CENP-E FL WT or CENP-E FL C2698A were infected with lentiviruses and selected with 1 μg/ml puromycin for 1 wk.

### siRNAs and transfection

The siRNAs targeting GAPDH (D-001830-01-05; Dharmacon), CENP-E (5′-CCA​CUA​GAG​UUG​AAA​GAU​A-3′; Kim et al., 2010), ZW10 (5′-UGA​UCA​AUG​UGC​UGU​UCA​A-3′; Sacristan et al., 2018), Spindly (5′-GAA​AGG​GUC​UCA​AAC​UGA​A-3′; Sacristan et al., 2018), Zwilch (5′-UCU​ACA​ACG​UGG​UGA​UAU​A-3′; Sacristan et al., 2018), and BUBR1 (5′-AGA​UCC​UGG​CUA​ACU​GUU​C -3′; Smith et al., 2019) were purchased from Dharmacon. RPE1 cells or HeLa FlpIn cells were transfected with siRNAs at 100 nM using the HiPerFect (Qiagen) according to the manufacturer’s instructions.

### Drug treatments on cells

RPE1 cells were treated with 6.6 μM nocodazole (M1404; Sigma-Aldrich) for 15 h (overnight) for fibrous corona formation before the fixation. HeLa FlpIn cells were treated with 6.6 μM nocodazole for 6∼8 h before the fixation. For cells transfected with siRNA, nocodazole was added 48 h after siRNA transfection. For the rescue experiments, 1 μg/ml doxycycline (D9891; Sigma-Aldrich) was added to cells 40 h after siRNA transfection to induce the expression of protein mutants. For nocodazole washout assay, 6.6 μM nocodazole was added to the culture medium for 15 h before washout. For CENP-E inhibition, cells were treated with 250 nM GSK923295 (S7090; Selleck Chem) for 15 h (overnight). For farnesyl transferase inhibition, cells were treated with 5 µm lonafarnib (S2797; Selleckchem) for 15 h (overnight). For the fast disassembly of microtubules, cells were treated with 13.2 μM nocodazole for 4 h. Cells were treated with 10 mM STLC (1291; Tocris Bioscience) for 15 h (overnight). For the experiments in which translocated fibrous corona proteins were released from spindle poles, cells were cotreated with STLC and GSK923295 for 15 h (overnight) and were further treated with 13.2 μM nocodazole for indicated time before fixation. In the TurboID pulldown experiment, HeLa cells were treated with 1 μg/ml doxycycline for 24 and 1 h of 250 μM biotin (B4501-5G; Sigma-Aldrich) before harvest. For spindle end-on attachment experiments, HeLa cells were cotreated with STLC and ZM447439 (2458; Tocris Bioscience) for 6 h before fixation.

### Antibodies

We used guinea pig polyclonal antibody against CENPC (PD030; MBL), and rabbit polyclonal antibodies against CENP-E (Brown et al., 1996), ZW10 (ab21582; Abcam), SPINDLY (A301-354A-1; Bethyl), ZWILCH (a gift from Andrea Musacchio, Max Planck Institute of Molecular Physiology), detyrosinated α -tubulin (ab48389; Abcam), astrin (A301-511A; Bethyl), BUBR1 (A300-995A; Bethyl), pericentrin (ab4448; Abcam), MAD2 (custom, B. O. 017), and ZWINT-1 (ab71982; Abcam). We used mouse monoclonal antibodies against α-tubulin (T9026; Sigma-Aldrich), MAD1 (MABE867; Merck Millipore), EB1 (610534; BD Biosciences), CENP-E (ab5093; Abcam), ZWILCH (a gift from Andrea Musacchio, Max Planck Institute of Molecular Physiology, Dortmund, Germany), p150^Glued^ (612708; BD Biosciences). We used the following secondary antibodies: Goat anti-guinea pig Alexa Fluor 647 (A21450; Invitrogen), Goat anti-rabbit Alexa Fluor 488 (A11034; Invitrogen), Goat anti-rabbit Alexa Fluor 568 (A11036; Invitrogen), Goat anti-rabbit Alexa Fluor 405 (A-31556; Invitrogen), Goat anti-mouse Alexa Fluor 568 (A11031; Invitrogen), Goat anti-mouse Alexa Fluor 647 (A21236; Invitrogen), and GFP-Booster Atto 488 (gba-488; Chromotek).

### Immunofluorescence staining

RPE1 or HeLa cells grown on 12-mm coverslips were fixed with −20°C methanol. After fixation, cells on coverslips were washed three times with PBS, followed by permeabilization with 0.1% triton X-100 in PBS for 2 min, then followed by washing three times in PBS supplemented with 0.05% Tween 20 (PBST), and were blocked with 2% BSA diluted in PBST for 30 min. After blocking, cells were incubated with primary antibodies diluted in PBST containing 2% BSA for 1 h at room temperature in humid conditions. Subsequently, cells were washed three times with PBST and incubated with secondary antibodies diluted in PBST together with or without DAPI for 1 h at room temperature. Then, cells were washed three times with PBST. In the end, coverslips were sequentially rinsed in 70% and 100% ethanol, air-dried, and mounted on glass slides with Prolong Gold antifade.

### Image acquisition and quantification

All images were acquired on a deconvolution system (DeltaVision Elite Applied Precision/GE Healthcare) with a 100×/1.40 NA UPlanSApo objective (Olympus) and imaging medium with *n* = 1.5160 (20130/20127-RCF; Cargille Laser Liquid) using SoftWorx 6.0 software (Applied Precision/GE Healthcare). Images were acquired as z-stacks at 0.2-μm intervals for 32 stacks using Photometrics Coolsnap HQ^2^ cameral (Roper Scientific) and deconvolved using SoftWoRx. The images are maximum-intensity projections of deconvoluted stacks.

Images were analyzed with Fiji (https://fiji.sc). For quantification of protein levels, all images of immunostaining experiments were acquired with identical illumination settings. Protein levels near the kinetochores were determined on maximum projections of z-stacks images using an ImageJ macro (Saurin et al., 2011; Wu et al., 2023) which thresholds the CENPC signal within the DAPI area or within the cytoplasm area in the images without DAPI staining. For quantification of EB1 levels on the kinetochores, the centrosome area was excluded as in a previous work (Wu et al., 2023). For fibrous corona size measurement, we determined Zwilch, MAD1, CENP-E, or ZW10 maximum projection area by an ImageJ macro threshold intensity of the protein of interest, as described previously (Wu et al., 2023).

### Live-cell imaging

For imaging of RPE1 cells coexpressing EB3-TagRFPT and GFP-Zwilch in nocodazole washout assay, RPE1 cells were cultured in a 24-well glass bottom plate (P24-0-N; Cellvis). Live-cell imaging was performed on a Nikon TiE microscope, controlled by NIS Elements software (v4.56; Nikon), by acquiring images every 20 or 30 s with z-stacks at 1-μm intervals for 15 stacks at 1  ×  1 binning on an Andor CSU-W1 spinning disk (50 μm disk) with Nikon 100× 1.45 NA oil objective and immersion oil with *n* = 1.528 (Applied Precision). 488 and 561 nm lasers were used for sample excitation and images were acquired using an Andor iXon-888 EMCCD camera. Nocodazole washout was done during the imaging with a warm culture medium. Before and after nocodazole washout, cells were kept at 37°C and 5% CO_2_ using a cage incubator and Boldline temperature/CO_2_ controller (OKO-Lab).

Imaging of HeLa FlpIn cells expressing H2B-mScarlet and HeLa FlpIn cells coexpressing LAP-CENP-E FL WT or LAP-CENP-E FL C2698A with H2B-mScarlet that were cultured in 8-well plates (μ-Slide 8 well, Ibidi) was performed on a Nikon TiE microscope, controlled by NIS Elements software (v4.56; Nikon), by acquiring images every 3 min with z-stacks at 2-μm intervals for eight stacks at 1  ×  1 binning on an Andor CSU-W1 spinning disk (50-μm disk) with Nikon 30× silicon objective and silicon immersion oil with *n* = 1.406 (SIL300CS-30CC; Olympus). 488 and 561 nm lasers were used for sample excitation and images were acquired using an Andor iXon-888 EMCCD camera. Cells were kept at 37°C and 5% CO_2_ using a cage incubator and Boldline temperature/CO_2_ controller (OKO-Lab).

### TurboID-based proteins pulldown for proteomics

In the TurboID-based proteins pulldown experiments, HeLa cells inducible expressing LAP-TurboID-CENP-E 2111-C mutants were treated with 1 μg/ml doxycycline for 24 h to induce the expression of LAP-TurboID-CENP-E 2111-C mutants and 1 h of 250 μM biotin before harvest. The cells were harvested by trypsinization and centrifuge. The cells were washed once with ice-cold PBS and lysated with lysis buffer (50 mM HEPES, pH 7.4, 150 mM NaCl, protease inhibitor, phosphatase inhibitor, 0.5% Triton X-100) on ice for 10 min. After lysis, the samples were centrifuged at 14,000 rpm for 20 min at 4°C. The supernatants of the centrifuged samples were incubated with the streptavidin magnetic beads (88816; Thermo Fisher Scientific) for 1 h in a 4°C cold room. The beads were washed with wash buffer (50 mM HEPES, pH 7.4, 150 mM NaCl, 0.1% Triton X-100) three times. The wash buffer was removed after the final wash and the beads were stored in a −80°C freezer.

### Liquid chromatography–mass spectrometry/mass spectrometry

Precipitated proteins were denatured and alkylated in 50 µl 8 M urea, 1 M ammonium bicarbonate (ABC) containing 10 mM tris (2-carboxyethyl) phosphine hydrochloride and 40 mM 2-chloro-acetamide for 30 min. After fourfold dilution with 1 M ABC and digestion with trypsin (20 µg/200 µl), peptides were separated from the beads and desalted with homemade C-18 stage tips (3 M), eluted with 80% acetonitrile (ACN) and, after evaporation of the solvent in the speedvac, redissolved in buffer A (0.1% formic acid [FA]). After separation on a 30-cm pico-tip column (75 µm ID; New Objective) in-house packed with C-18 material (1.9 µm aquapur gold, Dr. Maisch) using a 140 min gradient (7–80% ACN, 0.1% FA), delivered by an easy-nLC 1,000 (Thermo Fisher Scientific), peptides were electrosprayed directly into a Orbitrap Fusion Tribrid Mass Spectrometer (Thermo Fisher Scientific). The latter was set in data-dependent top speed mode with a cycle time of 1 s, in which the full scan over the 400–1,500 mass range was performed at a resolution of 240,000. Most intense ions (intensity threshold of 15,000 ions) were isolated by the quadrupole, where after they were fragmented with a higher-energy collisional dissociation collision energy of 30%. The maximum injection time of the ion trap was set to 50 ms with injection of ions for all available parallelizable time. Raw data were analyzed with MaxQuant (version 1.6.3.4) using the Homo Sapiens (taxonomy ID: 9606) fasta file, extracted from UniprotKB. To determine proteins of interest, the protein group output file was used to perform a differential enrichment analysis. Proteins with less than one unique peptide and proteins that have not been identified in at least two out of three of the replicates of one condition were filtered out. Then, a background correction and normalization of the data were performed by variance stabilizing transformation, shifting and scaling the protein intensities by sample group. A left-shifted Gaussian distribution randomization was used to impute since the data presented a pattern of missingness not at random. Finally, a differential enrichment analysis was performed to identify those proteins that were differentially enriched and selected those falling inside the threshold for log_2_ Fold Change and −log_10_ P value higher than 2. The program used for the analyses was R (version 4.0.4) through RStudio (version 1.5.64).

The mass spectrometry proteomics data have been deposited to the ProteomeXchange Consortium via the PRIDE (Perez-Riverol et al., 2022) partner repository with the dataset identifier PXD040338, where the data files are named as “emp” (=LAP-TurboID control), “WT” (=LAP-TurboID-CENP-E 2111-C WT), “C2689” (=LAP-TurboID-CENP-E 2111-C mutant C2698A), “519” (=LAP-TurboID-CENP-E 2,111–2,689), “517” (=LAP-TurboID-CENP-E 2111-C mutant C-10A9), and “513” (=LAP-TurboID-CENP-E 2111-C mutant C-10A7).

### Eukaryotic sequence database

The eukaryotic sequence database used for the evolutionary analysis consisted of 177 predicted proteomes derived from genomes or transcriptomes and spans a representative set of species across the eukaryotic tree of life. This dataset is derived from earlier work (Potter et al., 2023, *Preprint*), with some minor adjustments detailed in Stok et al. (2023).

### Homology detection and orthology assessment

Orthologs of CENP-E, SPINDLY, ROD, ZWILCH, and ZW10 were detected according to previously described workflows (Tromer et al., 2019; van Hooff et al., 2017, 2019). Briefly, iterative profile hidden Markov model–based (HMM) homology searches were performed on the eukaryotic sequence database using the “hmmsearch” method from the HMMER3 package v3.1b2 (Mistry et al., 2013). Here, we initially made use of previously published HMMs by our group (Tromer et al., 2019). Homologous sequences were aligned using MAFFT E-INS-i v.7.505 (Katoh and Standley, 2013), alignments were trimmed using trimAl v1.4 to only include alignment positions with at least 10% occupancy to remove gap-rich and poorly aligned regions (Capella-Gutiérrez et al., 2009), and finally, phylogenetic trees were inferred with FastTree v2.1.10 and IQ-Tree v2.2.0 to allow for phylogenomic assessment of homologous sequences (Minh et al., 2020; Price et al., 2010). In addition, we annotated putative orthologs using various homology-based methods including eggNOG-mapper v2 (Cantalapiedra et al., 2021), HHpred (Zimmermann et al., 2018), and BLASTP v2.9.0+ (Altschul et al., 1990). Besides detection of orthologous sequences in our current eukaryotic sequence database as described, we also included previously determined orthologs of fibrous corona components in Obazoan species not included in our initial selection of species to further increase the phylogenomic resolution in this clade based on (van Hooff et al., 2017). Combined, our methods allow for the establishment of fine-grained manually curated orthologous groups of fibrous corona components from our extended eukaryotic sequence database spanning the entire diversity of currently known eukaryotic life on Earth. Alignments and HMMs of the orthologous groups, as defined here, are available via the supplementary dataset accessible through https://figshare.com/articles/dataset/Supplementary_Dataset/24494893.

### Gene (re)prediction

As gene prediction is imperfect, we scrutinized absences or incomplete sequences inferred from previously published predicted proteomes using the workflow described previously by Tromer (2017). Briefly, we interrogate genome assemblies for regions homologous to a missing ortholog using TBLASTN (Altschul et al., 1990). Here, we use previously determined orthologs of closely related species as a query. If this method identified a homologous region in the genome, we performed gene prediction on this region using AUGUSTUS (Stanke and Morgenstern, 2005) and GENSCAN (Burge and Karlin, 1997) software using flexible settings to retrieve a set of possible transcripts, which were then assessed manually against ortholog alignments to determine whether this represents a previously not predicted ortholog of this gene in this species. (Re)predicted sequences of orthologs are indicated as such in the supplementary dataset, accessible through https://figshare.com/articles/dataset/Supplementary_Dataset/24494893.

### Statistical analysis

All data were analyzed by Excel for Mac (Version 16.77; Microsoft Office) and Prism 9 (Version 9.5.1; GraphPad Software). Data distribution was assumed to be normal but this was not formally tested. The results of Fig. 1, D and G; Fig. 2, C and F; Fig, 3, B, D, and F; Fig. 4, D and E; Fig. 5, F, G, I, J, and L; Fig. 6, B, D, E, G, I, and K; Fig. S1, A, C, E, G, J, K, M, and O; Fig. S2, C, E, G, and I; Fig. S3, H, I, J, and L; and Fig. S4, A–C, E, F, and I are presented as mean ± SD; error bars in figures represent SD for at least triplicate experiments. Paired two-sample two-tailed *t* test was used to analyze the statistical difference. Significance is indicated by P values.

### Online supplemental material

Fig. S1 shows that fibrous corona formation requires C-terminal farnesylation of CENP-E. Fig. S2 shows that farnesylated CENP-E is essential for kinetochore-derived microtubule nucleation. Fig. S3 shows that farnesylation promotes interaction of CENP-E with fibrous corona proteins. Fig. S4 shows that CENP-E impacts fibrous corona formation after initial RZZ-S kinetochore recruitment. Fig. S5 presents the comparative genomics of fibrous corona formation mechanisms in eukaryotes.

## Data availibility

The data generated are available in the published article and its online supplemental material. The mass spectrometry proteomics data have been deposited to the ProteomeXchange Consortium via the PRIDE partner repository with the dataset identifier PXD040338.
